# RAS activation induces synthetic lethality of MEK inhibition with mitochondrial oxidative metabolism in acute myeloid leukemia

**DOI:** 10.1038/s41375-022-01541-0

**Published:** 2022-03-30

**Authors:** Justine Decroocq, Rudy Birsen, Camille Montersino, Prasad Chaskar, Jordi Mano, Laury Poulain, Chloe Friedrich, Anne-Sophie Alary, Helene Guermouche, Ambrine Sahal, Guillemette Fouquet, Mathilde Gotanègre, Federico Simonetta, Sarah Mouche, Pierre Gestraud, Auriane Lescure, Elaine Del Nery, Claudie Bosc, Adrien Grenier, Fetta Mazed, Johanna Mondesir, Nicolas Chapuis, Liza Ho, Aicha Boughalem, Marc Lelorc’h, Camille Gobeaux, Michaela Fontenay, Christian Recher, Norbert Vey, Arnaud Guillé, Daniel Birnbaum, Olivier Hermine, Isabelle Radford-Weiss, Petros Tsantoulis, Yves Collette, Rémy Castellano, Jean-Emmanuel Sarry, Eric Pasmant, Didier Bouscary, Olivier Kosmider, Jerome Tamburini

**Affiliations:** 1 Université de Paris, Institut Cochin, CNRS UMR8104, INSERM U1016, F-75014 Paris, France; 2 Equipe Labellisée Ligue Nationale Contre le Cancer (LNCC), Paris, France; 3 Centre de Recherche en Cancérologie de Marseille (Cancer Research Center of Marseille.), CRCM, Inserm UMR1068, CNRS UMR7258, Aix Marseille Université U105, Institut Paoli Calmettes, Marseille, France; 4 Translational Research Centre in Onco-hematology, Faculty of Medicine, University of Geneva, and Swiss Cancer Center Leman, Geneva, Switzerland; 5 Hematology Laboratory, Assistance Publique-Hôpitaux de Paris, Centre-Université de Paris, Cochin Hospital, Paris, France; 6 Cancer Research Center of Toulouse, Unité Mixtes de Recherche 1037 INSERM, Toulouse, France; 7 Bioinformatics Platform- U900, Institut Curie, PSL Research University, Paris, France; 8 BioPhenics High-Content Screening Laboratory, Cell and Tissue Imaging Facility (PICT-IBiSA), Institut Curie, PSL Research University, Translational Research Department, Paris, France; 9 Pathology department, Geneva University Hospital, 1211 Geneva 4, Switzerland; 10 Cytogenetic Laboratory, Necker Hospital, Paris, France; 11 Hematology Department, Institut Paoli-Calmettes, Aix-Marseille Université, Marseille, France; 12 Inserm, CNRS, Institut Paoli-Calmettes, CRCM, Predictive Oncology, Aix-Marseille Université, Marseille, France; 13 Service d’Hématologie Adultes, Hôpital Universitaire Necker-Enfants Malades, Assistance Publique Hôpitaux de Paris, Paris, France; 14 Institut Imagine, INSERM U1163, 75015 Paris, France

**Keywords:** Acute myeloid leukaemia, Prognosis, Preclinical research

## Abstract

Despite recent advances in acute myeloid leukemia (AML) molecular characterization and targeted therapies, a majority of AML cases still lack therapeutically actionable targets. In 127 AML cases with unmet therapeutic needs, as defined by the exclusion of ELN favorable cases and of FLT3-ITD mutations, we identified 51 (40%) cases with alterations in RAS pathway genes (RAS+, mostly *NF1*, *NRAS*, *KRAS,* and *PTPN11* genes). In 79 homogeneously treated AML patients from this cohort, RAS+ status were associated with higher white blood cell count, higher LDH, and reduced survival. In AML models of oncogenic addiction to RAS-MEK signaling, the MEK inhibitor trametinib demonstrated antileukemic activity in vitro and in vivo. However, the efficacy of trametinib was heterogeneous in ex vivo cultures of primary RAS+ AML patient specimens. From repurposing drug screens in RAS-activated AML cells, we identified pyrvinium pamoate, an anti-helminthic agent efficiently inhibiting the growth of RAS+ primary AML cells ex vivo, preferentially in trametinib-resistant *PTPN11*- or *KRAS*-mutated samples. Metabolic and genetic complementarity between trametinib and pyrvinium pamoate translated into anti-AML synergy in vitro. Moreover, this combination inhibited the propagation of RA+ AML cells in vivo in mice, indicating a potential for future clinical development of this strategy in AML.

## Introduction

While acute myeloid leukemia (AML) is still associated with a low cure rate, recent advances in understanding its molecular complexity have significantly improved therapy for subgroups of patients, including those harboring *FLT3*, *IDH1*, or *IDH2* mutations [1]. However, more than half of AML cases still lack a druggable oncogenic target. Human cancers frequently harbor mutations in RAS oncogene family members, which drive oncogenesis by increasing cellular proliferation and survival. These are small protein GTPases, regulated by a switch between active GTP-linked and inactive GDP-bound states that is governed by a complex network of guanine exchange factors (GEFs, favoring RAS-GTP) and GTPase activating factors (GAPs, favoring RAS-GDP) [2]. RAS activation either due to extrinsic recruitment by transmembrane tyrosine kinase receptors or intrinsic mutations propagates through the downstream RAF-MEK-ERK and PI3K-AKT signaling pathways. Besides RAS-activating mutations that confer independence from physiological regulators, human cancers harbor mutations in other RAS network genes such as *NF1* (encoding neurofibromin, a RAS GAP)*, BRAF,* or *PTPN11* (encoding the SHP2 tyrosine phosphatase involved in RAS activation). Somatic alterations of RAS pathway genes, notably *NRAS*, *KRAS, PTPN11* (missense mutations), and *NF1* (mutations and deletions), are reported in up to 20% of AML cases [3].

Numerous studies in genetic mouse models of RAS-driven cancers have shown that RAS mutations are both early oncogenic events and essential for tumor maintenance. However, transformation of cells generally requires additional signals such as alterations in tumor suppressor genes that cooperate with RAS activation [2]. Recent gene essentiality profiling demonstrated that *NRAS* and *KRAS* mutations are driver oncogenes in AML, as attested by the dependency of RAS mutated AML cells on *NRAS* or *KRAS* expression, and by the discovery of synthetic lethal interactions between oncogenic RAS and effectors of RAS maturation or MAPK signaling that are exclusively found in RAS mutant AML cells [4]. The anticancer effect of genetic RAS suppression in RAS-driven mouse models and human cancer cell lines demonstrated that mutant RAS represents a bona fide therapeutic target in cancer. The recent paradigm shift achieved by the direct blocking of *KRAS* G12C mutation, as well as other strategies including downstream MAPK pathway inhibition show promise for the treatment of cancers dependent on RAS mutations, although signaling feedbacks, bypasses and tumor heterogeneity could limit their clinical efficacy [2, 5, 6].

We focused on AML cases with unmet prognostic and therapeutic needs, defined in our study as not belonging to the European leukemia network (ELN) favorable risk category [7], and being negative for FLT3-ITD mutation. From 127 AML cases, targeted next-generation sequencing revealed RAS pathway alterations (referred to as RAS+, mostly in *NF1*, *NRAS*, *KRAS*, *PTPN11*, and *CBL* genes) in 51 patients (40%). Among 79 intensively and homogeneously treated AML patients, RAS+ status correlated with higher clinical proliferation markers and reduced survival. While efficient against models of RAS-MAPK activation in vitro and in vivo, the MEK inhibitor trametinib had heterogeneous and modest activity against primary AML cells from patients ex vivo. From repurposing high-content pharmacological screens in RAS-activated AML cells, we identified pyrvinium pamoate, an anti-helminthic agent acting through inhibition of mitochondrial respiration, in our models. This compound impaired cell viability and colony formation in RAS+ primary AML samples with a more pronounced effect against trametinib-resistant cases. The combination of trametinib and pyrvinium pamoate was synergistic in vitro against AML cell lines and ex vivo in primary AML samples, and impaired the propagation of RAS mutated human AML cells in vivo in mice. These results highlighted the translational opportunity in developing pyrvinium pamoate for RAS-activated AML.

## Material and methods

### Patients

AML patients provided written informed consent (IRB Ile de France II: 2015-08-11-DC) in accordance with the declaration of Helsinki. AML patient database was declared to the CNIL (MR-4 French standard, registration number 2214849v 0). Blood or bone marrow samples were submitted to a Ficoll-Hypaque density gradient (1800 rpm during 30 mn) as previously described [8]. Mononuclear cells stored in the Hematology Cell Biobank of Cochin Hospital were collected, washed once in phosphate buffer saline (PBS), then incubated with a red cell lysis buffer (155 mM NH_4_Cl, 10 mM KHCO_3_, 0.1 mM EDTA) for 5 min and washed once again in PBS. DNA was immediately extracted using the AllPrep DNA/RNA mini Kit (80204, Qiagen, Hilden, Germany) according to manufacturer’s procedures while leftover cells were cryopreserved. RNA and proteins were extracted from cryopreserved cells shortly after thawing using AllPrep DNA/RNA/Protein Mini Kit (80004, Qiagen) according to manufacturer’s instructions. Samples containing less than 70% blast cells before Ficoll were either purified using MiniMACS immunoaffinity columns (Miltenyi Biotec, Cologne, Germany) in case of CD34 membrane expression, or sorted with a BD FACSAria™ III cell sorter (BD Biosciences, Franklin Lakes, USA) gating the low side scatter and low CD45-expressing population.

### DNA sequencing

We used two complementary approaches based on AmpliSeq^TM^ panels designed for Ion Torrent^TM^ (Thermo-Fischer Scientific, Waltham, USA), as reported [9] and Illumina (AmpliSeq for Illumina Myeloid Panel, Illumina, San Diego, USA) technologies. More specifically, we used two customized AmpliSeqTM panels to screen mutations in *NF1*, *EED*, *EZH2*, and *SUZ12* (NF1/PRC2 panel) and in 30 RAS pathway genes (RAS panel). These two panels were designed using AmpliSeq^TM^ Designer (version 4.47) on Human genome hg19, and sequencing was performed on Ion PGM™ (Life Technologies, Carlsbad, USA) on a dedicated 318 V2 chip, as reported [10, 11].

### Gene expression profiling

#### Microarrays

RNA quality was evaluated with a Bioanalyzer 2100 (using Agilent RNA6000 nano chip kit, Santa Clara, USA), and 100 ng of total RNA was reverse transcribed using the GeneChip^®^ WT Plus Reagent Kit according to the manufacturer’s instructions (Affymetrix, Thermo Fischer Scientific). Raw fluorescence intensities were normalized and analyzed as reported [8]. Raw data are available at ArrayExpress with the accession number E-MTAB-10261.

#### RNA sequencing

RNA sequencing library preparation was performed using Illumina TruSeq stranded mRNA protocol. The average number of per sample reads was 65 × 10^6^. We mapped the reads to the human genome reference “hg38_chr_only_and_herpes.fa” using STAR aligner (STAR v2.6.1a). Base-calling accuracy scores (Phred quality score, Q30) were obtained from demultiplexer bcl2fastq v2.20. Detailed methods for RNA-seq quantification and differential gene expression analysis are provided in the Supplemental Material. Raw data are available at ArrayExpress under the accession number E-MTAB-10048.

### Fluorescence in situ hybridization (FISH)

Dual-color FISH experiments were performed using a XL TP53/NF1 D-5089-100-OG probe (Metasystems probes, Altlussheim, Germany), targeting a 167 kb region of *TP53* (probe labeled with Rhodamine-dUTP) and a 312 kb region of *NF1* (probe labeled with FITC-dUTP). Hybridization was performed as previously described [12]. The images were captured by a CCD camera fixed on a BX61 microscope (Olympus, Rungis, France), and processed with a Case data Manager 6.0 software (Applied Spectral Imaging, Carlsbad, USA).

### Cell lines and reagents

We used a panel of AML cell lines (Supplementary Table 1) which were identified by PCR-single-locus-technology (Promega, PowerPlex21 PCR Kit, Eurofins Genomics, Luxembourg). Cells were cultured in RPMI 1640 medium (Gibco 61870; Life Technologies) supplemented with 10% FCS, 2 mM glutamine (Gibco 25030; Life Technologies), 100 IU/mL penicillin and 100 µg/mL streptomycin (Gibco 15140; Life Technologies) at 37 °C under a 5% CO_2_ atmosphere. TF-1 cells were cultured with 5 ng/mL of human GM-CSF (130-093-866, Cologne, Germany). The murine hematopoietic BaF/3 cells were cultured with IL-3 provided by a conditioned medium harvested from cultured WEHI-3 cells [13]. Other cell lines were grown without cytokines. Trametinib was purchased from Selleck chemicals LLC (Houston, USA) and Pyrvinium pamoate was from Sigma Aldrich. The target selective inhibitor library (L3500) was obtained from Selleck chemicals LLC. A unique collection of 1280 off-patent small molecules (PCL, mostly drugs approved by FDA, EMA, and other agencies) was obtained from Prestwick Chemicals V3.

**Table 1:** Comparison of clinical characteristics of 79 intensively treated AML patients dependent on the presence of RAS pathway mutations.

	RAS+	RAS−	*p*-value	Test
N	34	45		
Male (%)	22 (65%)	29 (64%)	>0.999	Fischer exact
Age (years)	61 (23–78)	61 (26–82)	0.8	Student
WBC (×10^9^/l)	19.5 (1.1-287)	2.3 (0.8-170)	0.0002	Mann-Whitney
missing	1	1		
% blast	62 (22–94)	44 (7–98)	0.013	Mann-Whitney
missing	1	3		
LDH (UI/l)	501 (150–3516)	347 (56–18,000)	0.0236	Mann-Whitney
missing	0	3		
ELN 2017				
INT	9 (26.5%)	19 (73.5%)	0.1632	Mann-Whitney
ADV	25 (42.2%)	26 (57.8%)		Mann-Whitney
Allo (%)	8 (24%)	13 (32%)	0.6188	Fischer exact
missing	0	4		
Refractory (%)	13 (43%)	17 (39%)	0.5046	Fischer exact
NE	4	2		

*N* number of cases, *WBC* white blood cell count, *missing* missing data, *% blast* percentage of bone marrow blast cells, *ELN 2017* 2017 European leukemia network risk stratification, *INT* intermediate, *ADV* adverse, *Allo* allogenic stem cell transplantation, *NE* not evaluable.

### Constructs

#### CRISPR/Cas9

Human and murine *NF1*-targeting guide RNA were designed using the Optimized Crispr Design application from the laboratory of Dr Feng Zhang (http://crispr.mit.edu/, no longer available) as previously described [8]. The sequences used were (forward and reverse):

- human NF1^KO_1^: CACCGTTGTGCTCAGTACTGACTT and AAACAAGTCAGTACTGAGCACAAC
- human NF1^KO_2^: CACCGAGTCAGTACTGAGCACAACA and AAACTGTTGTGCTCAGTACTGACTC
- human control: CACCGTAGGCGCGCCGCTCTCTAC and AAACGTAGAGAGCGGCGCGCCTAC
- mouse NF1^KO_1^: CACCGCTCGTCGAAGCGGCTGACCA and AAACTGGTCAGCCGCTTCGACGAGC
- mouse NF1^KO_2^: CACCGCAGATGAGCCGCCACATCGA and AAACTCGATGTGGCGGCTCATCTGC

The human guides were then cloned into the plentiCRISPRv1 puromycin plasmid (#49535 no longer available, Addgene) [14] while the murine guides were cloned into the plentiCRISPRV2 mCherry plasmid (LentiCRISPRv2-mCherry was a gift from Agata Smogorzewska (Addgene plasmid # 99154; http://n2t.net/addgene:99154; RRID:Addgene_99154).

#### Expression vectors

Hs.NRAS G12D pDonor 255 (gift from Dominic Esposito, Addgene plasmid # 83176; http://n2t.net/addgene:83176; RRID:Addgene_83176) was cloned into the plenti PGK puro DEST w529-2 (gift from Eric Campeau and Paul Kaufman, Addgene plasmid # 19068; http://n2t.net/addgene:19068; RRID:Addgene_19068) [15] using the Gateway system (Life Technologies, Carlsbad, CA, USA). *PTPN11* WT and D61Y plasmids were generated using the GeneArt gene synthesis technology (ThermoFischer Scientific).

### Immunoblots

Cells were lysed in 100 μL 1× Laemmli buffer [62.5 mM Tris HCl pH 6.7, 10% glycerol, 2% sodium dodecylsulfate (SDS), 24 mM dithiotreitol, 2 mM Vanadate, bromophenol blue], heated at 90 °C for 5 min and resolved by SDS-polyacrylamide gels electrophoresis, transferred to nitrocellulose membranes, and probed with primary antibodies. Protein signals were revealed by chemoluminescence (ECL, Bio-Rad, Marnes la coquette, France) and detected using a CCD camera (LAS 3000 Fujifilm, Tokyo, Japan). The following antibodies were used: phospho-ERK 1/2 T202-Y204 (#4377), ERK (#9108), NF1 (#14623), Cleaved caspase 3 (#9661), and PARP (#9542) from Cell Signaling Technology, RAS (05-016) from Merk Millipore and β-actin (A1978) from Sigma Aldrich.

### RAS pull down assay

RAS activity was assessed by a GST-RAF1-RBD pull-down assay according to manufacturer’s instruction (Merck Millipore, Burlington, USA). Briefly, 5 × 10^6^ cells were lysed and active RAS was pulled down after interaction with a RAF1-RBD motif conjugated with agarose beads. Beads were then solubilized in Laemmli buffer and RAS detection—proportional to its activity quantified by the RAS-RAF interaction—was performed by immunoblotting.

### Mouse experiments

#### Cell line derived Xenografts

All animal experiments were performed in compliance with the laws and in agreement with the French Guidelines for animal handling and approved by animal ethics committees. Healthy 6–9-weeks-old NOD.Cg-Prkdc scid/J mice (NSG) were maintained under sterile conditions with sterilized food and water provided ad libitum and maintained on a 12 h light and 12 h dark cycle. In experiments with TF-1 cells, mice were preconditioned with 20 mg/kg Busulfan (Pierre Fabre, France) and injected via tail vein with 2 × 10^7^ viable cells in 200 μl sterile PBS. In other experiments, mice were injected via tail vein with 0.2 × 10^6^ viable luciferase-expressing HL-60 cells in 100 μl sterile PBS. The day after cells transplant, animals were randomly assigned into treatment groups. Trametinib and pyrvinium were given by oral gavage in 4% DMSO corn oil, and by intraperitoneal injection in 5% DMSO/PBS, at 0.25 mg/kg/d and 0.25 mg/kg/d, respectively, 5d/week. Bioluminescence analysis was performed using PhotonIMAGER OPTIMA (Biospace Lab, France) following addition of endotoxin-free luciferin (30 mg/kg).

#### Patient derived Xenograft

We xenografted a RAS+ patient-derived AML sample to NSG mice. We injected 5 × 10^5^ cells in 100 µL of PBS in the tail vein of each mice. After the detection by flow cytometry of human CD33+AML cells in peripheral blood, mice were treated with Vehicle (*N* = 8), Trametinib (*N* = 9), Pyrvinium (*N* = 9) or trametinib and Pyrvinium combination (*N* = 9) by oral gavage 5 days a week. Doses used were 0.25 mg/kg/d trametinib (in 5% DMSO PBS), 0.5 mg/kg/d pyrvinium (in 4% DMSO corn oil), combination of 0.25 mg/d trametinib and 0.25 mg/kg/d pyrvinium (initially 0.5 mg/kg/d dropped to 0.25 mg/kg/d after the first two doses), or combination of the vehicles in the control group. Disease propagation was monitored by hCD33 detection in peripheral blood, and AML tumor burden was quantified after sacrifice by hCD33 detection in whole bone marrow and spleen of each mice. Investigators were not blinded to the group allocation during experiment, but were blinded when assessing the outcome.

### Cell viability assays

#### Uptiblue^®^

Cells were seeded in 100 μl of culture medium in 96 well plates for 48 h. Ideal cell density was determined to be 2 × 10^4^/ml and 10^6^/ml for cell lines and primary AML cells, respectively. The UptiBlue^®^ viable cell-counting reagent (Interchim, Montluçon, France) was then added for 4 h and fluorescence was measured with a Typhoon 8600 scanner (GE Healthcare Bio-Sciences, Buc, France).

#### CellTiter-Glo^®^ 2.0 assay

Cells were seeded in at 1.25 × 10^5^/ml in 40 μl culture medium in 384-well plates for 24 h, then 10 μl of culture medium without or with compound were added for 72 h. At the end of incubation, 25μl of the CellTiter-Glo® 2.0 Assay reagent (Promega Inc., Madison, USA) was added in each well. The contents were mixed for 2 min at 300 rpm on an orbital shaker (Titramax 100, Dutscher, Issy-les-moulineaux, France) and plates were incubated for 10 min at room temperature to stabilize luminescent signals. Units of luminescent signal generated by a thermo-stable luciferase are proportional to the amount of ATP presented in viable cells. Luminescence was recorded using a CLARIOStar (BMG Labtech, Ortenberg, Germany) reader at a gain of 3600.

### Pharmacological screening

#### Target selective inhibitor library screen

Compounds from the target selective inhibitor library were solubilized in DMSO, and distributed at the final concentration of 10 μM to cells seeded in 96-wells plates. Cell viability was assessed after 48 h using the Uptiblue^®^ reagent. Experiments were repeated three times separately and, after background noise subtraction, outliers removal and data normalization, results were presented as a percentage of each condition relative to the control (DMSO) condition.

#### Prestwick chemical library^®^ (PCL) screen

Cells were seeded in 384-well plates (ViewPlate-384 Black - PerkinElmer, ref. 6007460) using a MultiDrop combi (ThermoFisher Scientific), in 40 μL of cell media at 37 °C for 24 h. Optimal cell densities were determined as 5 × 10^3^ for CTR and NF1^KO_1^ and 6 × 10^3^ for NF1^KO_2^ TF-1 cell lines using T4 Cellometer (Nexcelom). Detailed procedures and data processing methods are provided in the Supplementary Material section.

### Bioenergetic analysis assays

Oxygen consumption rate (OCR) was measured using a Seahorse XF96 extracellular flux analyzer (Seahorse Bioscience, North Billerica, MA, USA). Briefly, 1.5 × 10^5^ cells were seeded in 96-well XF96 well plates coated with BD Cell-Tak (Becton Dickinson Biosciences, Franklin Lakes, NJ, USA) and loaded with XF Dulbecco’s Modified Eagle’s Medium. After 1 h incubation at 37 °C without CO_2_, cells were transferred to the XF96 analyzer. Oligomycin (1 µM) was added after 20 min, followed by FCCP (2 µM) after 40 min and Antimycin A/Rotenone (1 µM) after 59 min.

### Leukemia colony forming units (L-CFU) assay

L-CFU assays were performed as previously described [16]. Briefly, AML cells were seeded at 10^5^/ml in H4230 medium (StemCell Technologies, Vancouver, Canada) supplemented with 10% of conditioned medium harvested from cultured 5637 cells. At day 7, L-CFU (colony of >20 cells) were scored under an inverted microscope.

### Statistics

In event-free survival (EFS) analysis, events were defined as induction failure, relapse or death of any cause and data were not censored at the time of stem cell transplantation [17]. Death of any cause accounted for events in overall survival (OS) analysis. Statistical analysis of categorical variables was performed using the Fisher exact test. Univariate and multivariate analysis of survival were performed with Log Rank (Mantel Cox) test and Cox multiple regression model, respectively, using the survival R package. Differences between the mean values obtained for the experimental groups were analyzed using the two-tailed Student’s *t* test or a Mann-Withney test for parametric and nonparametric data, respectively. Two-by-two comparisons between colonies ratio of matched-pairs samples were made by using a Wilcoxon matched-pairs signed rank test. Statistical analyses were performed using Prism software 8.1.1 (GraphPad). Vertical bars indicate standard deviations. *P < 0.05, **P < 0.01, ***P < 0.001.

## Results

### Landscape and clinical correlations of RAS pathway alterations in AML

**Fig. 1:**
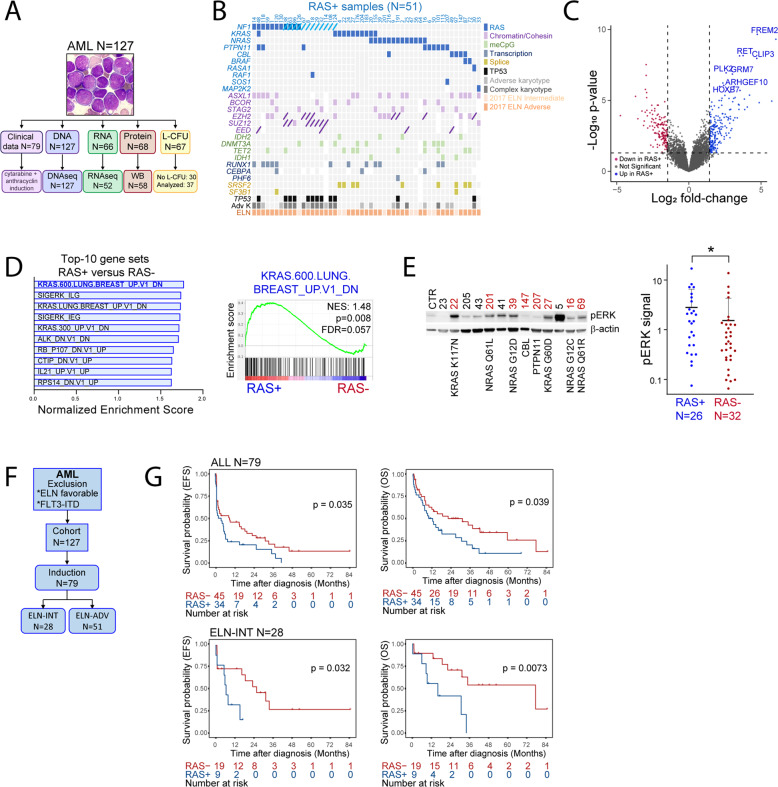
Landscape and clinical correlations of RAS pathway alterations in AML. **A** From 127 primary AML samples, we obtained DNA, RNA and protein samples in 127, 66, and 68 cases, respectively. After quality check, 127, 52, and 58 specimens were used for DNA sequencing (DNAseq), RNA sequencing (RNAseq) and Western blots (WB), respectively. L-CFU assays were performed in 69 cases, among which 37 generated more than five colonies (Analyzed samples). Clinical records were obtained in 79 cases having received an intensive chemotherapy regimen (cytarabine + anthracyclin induction). **B** Next-generation sequencing (NGS) in 51 RAS+ diagnosis samples of AML patients. RAS+ mutations (in blue) are represented when detected in at least one sample, and myeloid neoplasm-related mutations when occurring in at least five samples, regardless the number of mutations detected for a given gene. Variants are indicated by colored squares and deletions by an inclined line. Molecular categories are represented by a color code as indicated in the legend. Cytogenetics (complex and/or adverse karyotype versus others) and European leukemia network 2017 (ELN) risk category (intermediate or adverse) are indicated. **C** Gene expression profiling by RNA sequencing in 58 AML cases using a volcano plot representation of differential gene expression between RAS+ and RAS− cases. Genes with significant differential expression (cutoff fold-change = 1.5 and *p*-value 0.05) are highlighted in blue (up in RAS+ ) or in red (up in RAS−), and the names of RAS−related genes among the top-20 differentially expressed between RAS+ and RAS− samples are provided. **D** Left panel: top-10 gene sets significantly enriched in the transcriptomic analysis of RAS+ versus RAS− samples using the oncogenic signature gene set from GSEA. Right panel: representative enrichment plot of the KRAS.600 signature. **E** Protein extracts from 58 AML samples were analyzed by Western blot for ERK phosphorylation. Left panel: representative Western blots with RAS+ cases indicated in red along with type of mutation. CTR indicate a control sample (from an AML patient) used for normalization across Western blots. Right panel: quantification of phospho-ERK relative to β-actin (used as loading control) signal intensities. Ratio obtained for each sample (26 RAS+ and 32 RAS−) was normalized to the ratio obtained for the CTR sample for each membrane, separately. **F** After exclusion of ELN favorable risk category and FLT3-ITD positive cases, 127 AML cases were included, and among them 79 (62%) were homogeneously treated by an anthracycline- and cytarabine-based induction chemotherapy, including 28 and 51 from the ELN intermediate (INT) and adverse (ADV) risk category. **G** Event-free and overall survival (EFS, left panels and OS, right panels, respectively) of the whole cohort of intensively treated AML patients (*N* = 79, upper panels) and of ELN INT patients (*N* = 28, lower panels) dependent on RAS+ status.

In a cohort of 127 AML patients with unmet need for prognostic markers and new efficient therapies, we obtained DNA, RNA and protein samples in 127, 52 and 58 cases, respectively, and collected clinical data in 79 patients homogeneously treated with intensive induction therapy (Fig. 1A). To constitute our cohort, we excluded cases of the 2017 ELN favorable risk group (Supplementary Fig. 1A), and those harboring *FLT3* internal tandem duplication (FLT3-ITD) mutations, as mutually exclusive of RAS pathways alterations at diagnosis (Supplementary Fig. 1B), even if *NRAS* and *KRAS* mutations are frequently detected in relapse samples after FLT3-targeted tyrosine kinase inhibitor therapy [18].

Using targeted next-generation sequencing (NGS), we detected at least one RAS pathway gene alteration in 51 (40%) AML samples of 127 samples tested (Fig. 1B and Supplementary Fig. 1C). *NF1* mutations or deletions were found in seven and eight cases, respectively, and co-occurred in four (15% of cases had *NF1* alteration), while *NRAS, KRAS, PTPN11, CBL,* and *BRAF* mutations were detected in 13 (10.4%), 11 (8.6%), 9 (7.2%), 5 (4%), and 2 (1.6%) cases, respectively (Fig. 1B and Supplementary Fig. 1D, left panel). *RAF1, RASA1, SOS1,* and *MAP2K2* mutations were detected in a single case each in our cohort. We observed a co-occurrence of RAS pathway mutations in ten samples, mostly concerning *NRAS, KRAS* and *PTPN11* genes (Supplementary Fig. 1D, middle panel). In contrast to other RAS pathway mutations, *NF1* mutations or deletions were frequently associated to complex cytogenetics and *TP53* mutations (Fig. 1B). However, 3/12 *NF1* deletions (25%) were detected in the absence of complex cytogenetic abnormalities, two by fluorescence in situ hybridization (FISH) and one by NGS (Supplementary Fig. 1D, right panel). We also sequenced the polycomb complex repressor 2 (PRC2) components *EZH2*, *SUZ12*, and *EED*, as frequently co-altered with RAS pathway genes in other cancers [19] in 104 samples from our cohort, and found that 50% and 13.8% of RAS+ and RAS− cases, respectively had concomitant PRC2 alteration (*p* < 0.0001, Supplementary Fig. 1E). Variant allele frequencies allowed generating hypothesis on clonal architecture in 41 AML samples, and we estimated that RAS pathway mutations were clonal and sub-clonal in 10 and 31 cases, respectively (Supplementary Fig. 1F). Moreover, the repartition of AML-related mutations and of complex karyotype was similar between the RAS+ and RAS− groups, except for *IDH2* mutations more frequently detected in RAS− patients (Supplementary Table 2). Together these results provided a genetic landscape of RAS+ AML in a selected cohort of patients with unmet prognostic and therapeutic needs.

**Table 2:** Multivariate analysis.

We first performed a univariate Cox regression analysis in the whole cohort (*N* = 79, referred to as ALL) or in the ELN INT subgroup (*N* = 28) on the following parameters: age, sex, percentage of bone marrow blast cells, WBC, LDH levels, ELN 2017 status, RAS status, response to induction, allogenic stem cell transplantation. Then we applied a multivariate Cox model to the variables reaching significance in the univariate analysis.

We performed RNA sequencing (RNAseq) in 52 samples (26 RAS+ and 26 RAS−). The distribution of mutations commonly found in myeloid neoplasm was similar between RAS+ and RAS− samples (Supplementary Table 2). Differential gene expression analysis showed that top upregulated genes were frequently connected to RAS pathways, and that an enrichment in RAS-related gene sets was found in RAS+ cases (Fig. 1C, D and Supplementary Fig. 1G). We also analyzed 145 cases from the TCGA database with available RNAseq data, and observed a similar enrichment in RAS−related gene sets in RAS+ cases (Supplementary Fig. 1H). However, RAS+ samples did not delineate a specific cluster (Supplementary Fig. 1I), suggesting that the impact of RAS activation on transcription is limited compared to other molecular drivers in AML, as reported [20]. We investigated ERK phosphorylation status as a readout of RAS activity in protein extracts from 58 AML samples (including 26 and 32 RAS+ and RAS− cases, respectively), and observed that ERK phosphorylation was higher in RAS+ samples (Fig. 1E). These data showed that molecular features of RAS activation were detected in RAS+ primary AML samples.

We analyzed the clinical and biological characteristics of 79 patients from our cohort homogeneously treated with cytarabine- and anthracycline-based induction chemotherapy (Fig. 1F). While gender and age, as well as the proportion of secondary AML were homogeneously distributed, RAS+ patients had a significantly higher white blood cell count (WBC), percentage of bone marrow-infiltrating blast cells and lactate dehydrogenase (LDH) levels (Table 1). Notably, both groups had the same proportion of refractory disease and completion of allogenic hematopoietic stem cell transplantation (Table 1). Univariate analysis revealed that age over 60, WBC over 50 × 10^9^/l, high LDH levels, and ELN adverse risk category were associated with a reduced survival probability (Table 2). Moreover, RAS+ status was significantly associated with reduced event-free and overall survival (EFS and OS, respectively) probabilities, and more markedly in the ELN intermediate (ELN_INT) risk category (Fig. 1G). The adverse prognostic impact of RAS+ status on OS remained significant in the ELN-INT group but not in the whole cohort when analyzed using a multiple regression model (Table 2). When analyzed together, *NRAS* and *KRAS* mutations had no significant prognostic impact in our cohort, whereas *KRAS* mutations negatively influenced survival when considered individually (Supplementary Fig. 1J). From the BEAT AML cohort data [21], we found 92 ELN-INT AML cases also fulfilling our inclusion criteria and treated with intensive chemotherapy. While RAS pathway mutations were less frequently detected in this cohort (18 cases, 19.5%), we observed a tendency toward an association between RAS+ status and adverse outcome (Supplementary Fig. 1K).

These data suggested that RAS pathway alterations were associated with increased proliferation markers, and associated with reduced survival probability in selected AML cases, particularly within the ELN intermediate group.

### Modelization of RAS pathway gene alterations revealed oncogenic addiction in AML

**Fig. 2:**
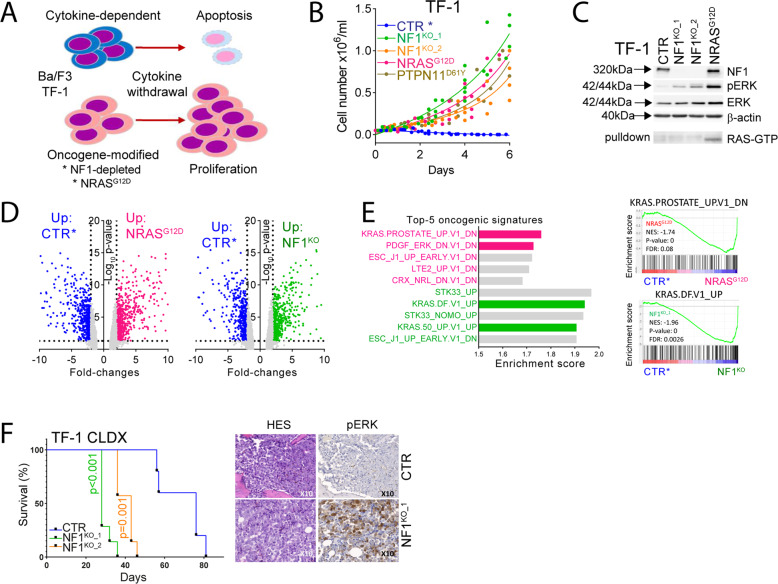
Modelization of RAS pathway gene alterations revealed oncogenic addiction in AML. **A** Schematic representation of cytokine-dependent cell lines models. TF-1 and Ba/F3 cell lines are cytokine-dependent (GM-CSF and IL-3, respectively). Cytokine starvation induces cell-cycle arrest and apoptosis in these cells. Activation of oncogenic pathways—such as RAS signaling—allow cytokine-independent growth of these cells. TF-1 cells were genetically modified to induce RAS activation. Two different guides of NF1-targeting CRISPR/Cas9 (NF1^KO_1^ and NF1^KO_2^), *NRAS*^G12D^, and *PTPN11*^D61Y^ constructs were transduced to TF-1 cells using lentivirus. Control cells (CTR) were transduced with a lentivirus expressing a non-targeting sgRNA. **B** Cell counting during six days after cytokine starvation in the modified TF-1 cell lines. **C** Immunoblotting of total cell lysates and of RAS-RAF1 pulldowns from CTR, NF1^KO_1^, NF1^KO_2^ and NRAS^G12D^ TF-1 cell lines with antibodies directed against NF1, phospho-ERK, ERK, RAS and β-actin. Black arrows indicate molecular weight of each proteins. Gene expression profiling by microarrays in CTR, NF1^KO_1^, and NRAS^G12D^ TF-1 cells. **D** Volcano plot representation of differential gene expression between control (cultured with GM-CSF, as indicated by *, in blue) and NRAS^G12D^ (left panel, in pink) or NF1^KO_1^ (right panel, in green). **E** Left panel: representation of the top-5 most significantly enriched gene expression signatures in the NRAS^G12D^ (in pink) or NF1^KO_1^ (in green) versus CTR* comparisons. Right panel: representative enrichment plots. **F** NOD/SCID gamma-null (NSG) mice were xenografted with 2 × 10^6^ cells from the CTR, NF1^KO_1^ or NF1^KO_2^ TF-1 cell lines. Left panel. Survival curves of the CTR (*N* = 5), NF1^KO_1^ (*N* = 7) and NF1^KO_2^ (*N* = 7) groups. Right panel. Hematoxylin-eosin staining (HES) and phospho-ERK immunohistochemistry (IHC) of paraffin-embedded bone marrow samples from those mice. Images were captured using the slide scanner and software Zeiss Axioscan.Z1 (Carl Zeiss AG) at a tenfold magnification (×10).

We used cytokine-dependent cell lines to investigate the oncogenic potential of RAS pathway genetic modifications [4]. TF-1 are human AML cell line requiring granulocyte-macrophage colony stimulating factor (GM-CSF) to proliferate and survive in vitro. The Ba/F3 murine cell line established from normal pro-B cells is dependent on interleukine-3 (IL-3). After cytokine starvation, parental cell lines undergo cell cycle arrest and apoptosis, while cells modified with an oncogenic signal exponentially grow in the absence of cytokines (Fig. 2A).

We used CRISPR/Cas9 and two different *NF1* sgRNAs to deplete neurofibromin (NF1^KO_1^ and NF1^KO_2^), and expressed mutant *NRAS*^G12D^ and *PTPN11*^D61Y^ in TF-1 and Ba/F3 cells. In following experiments, the genetic background from these isogenic cell lines remained stable and the differences between parental and modified cells could be directly attributed to RAS dependency. After lentiviral transduction, cells were starved from cytokines, and NF1^KO^ and NRAS^G12D^ cells grew readily from a bulk population in both TF-1 and Ba/F3 cell lines in contrast to control cells, while *PTPN11*^D61Y^ expression led to cytokine-independent growth in TF-1 cells only (Fig. 2B and Supplementary Fig. 2A). RAS activation was detected in NF1^KO^ and *NRAS*^G12D^ cells compared to controls (CTR) by increased ERK phosphorylation and active RAS form detection in RAS−RAF1 pulldown assays (Fig. 2C and Supplementary Fig. 2B).

We selected NF1^KO_1^ and NRAS^G12D^ to perform transcriptomic assays, and not PTPN11^D61Y^ as this construct less robustly activated MEK and ERK signaling in TF-1 cells (Supplementary Fig. 2C). After 6 h of GM-CSF starvation, we observed that many genes were differentially regulated between the CTR and RAS-activated conditions (Fig. 2D). Moreover, we found that RAS-activation signatures scored among the most significantly enriched both in NF1^KO_1^ and NRAS^G12D^ compared to CTR conditions (Fig. 2E). TF-1 cells experience a erythroid differentiation when cultured with EPO [22], and we observed hemoglobinization of CTR but not NF1^KO^ cells in long-term culture with EPO, suggesting that RAS activation blocked the differentiation program to favor proliferation (Supplementary Fig. 2D).

We investigated the oncogenic potential of RAS activation in vivo in NOD/SCID gamma-null (NSG) mice using CTR and NF1^KO^ TF-1 cells lines, but not NRAS^G12D^ mutant, which represents an established model of myeloid neoplasms propagation in mice dependent on MEK activity [23, 24]. Xenografted mice developed AML-related symptoms within a median time of 28 days, 43 days, and 76 days for NF1^KO_1^, NF1^KO_2^, and CTR groups, respectively (*p* < 0.001 for comparison between NF1^KO^ and control cells, Fig. 2F, left panel). Staining of bone marrow sections showed an increased ERK phosphorylation in mice transplanted with NF1^KO^ cells, in agreement with our in vitro observations (Fig. 2F, right panel). Together these results suggested that *NF1* suppression and *NRAS*^G12D^ expression led to RAS/MAPK activation and dependency in AML models.

### Heterogeneous activity of MEK inhibitors against RAS+ AML

**Fig. 3:**
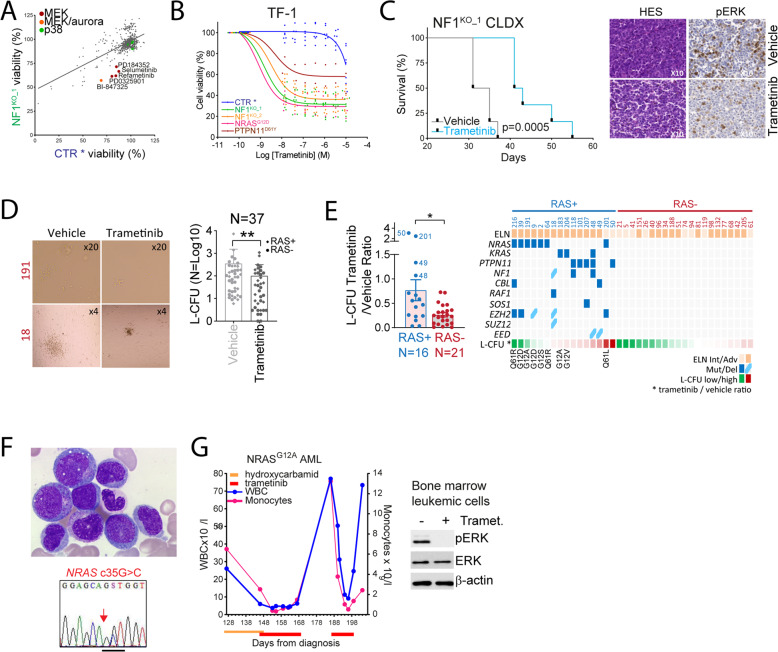
Heterogeneous activity of MEK inhibitors against RAS+ AML. **A** We applied the target selective inhibitors library (592 unique compounds) to CTR and NF1^KO_1^ TF-1 cells for 48 h, before cell viability quantification using the uptiblue fluorescent reagent. Each dot on the graph represents the mean of three independent experiments. **B** Dose–response curves of the MEK inhibitor trametinib (from 10^−6^ to 13.7 × 10^−9^ M) on CTR, NF1^KO_1^, NF1^KO_2^, NRAS^G12D^, PTPN11^WT^, and PTPN11^D61Y^ TF-1 cell lines. CTR and PTPN11^WT^ cells were cultured with GM-CSF as indicated by *, while the remaining were not, and cell viability was measured by the uptiblue reagent. **C** NSG mice were xenografted with 2 × 10^6^ cells from the NF1^KO_1^ TF-1 cell line (CLDX: cell line-derived xenograft) and vehicle or 0.5 mg/kg/d trametinib were given daily by oral gavage from day 8 post-transplant. Left panel: survival curves of this experiment (*N* = 6 in each group). Leukemia colony forming units (L-CFU) assays of primary samples from AML patients incubated with vehicle or 50 nM trametinib for 7 days. **D** Left panel: representative pictures of L-CFU in RAS+ (numbers in red) or RAS− samples at an ×4 or ×20 magnification as indicated. Right panel: comparison of the L-CFU number between vehicle- and trametinib-treated samples (*N* = 39). RAS+ and RAS− samples are identified by a diamond and a circle, respectively. **E** Left panel: histograms represent L-CFU ratio between trametinib and vehicle conditions in RAS+ and RAS− samples (*N* = 16 and 23, respectively). Right panel: representation of the L-CFU assays results dependent on *NRAS*, *KRAS*, *PTPN11*, *NF1*, and *CBL* mutational status. Type of amino acid substitutions are provided for *NRAS* and *KRAS* mutations. Description of the case of a NRAS^G12A^-mutated AML patient treated with trametinib. **F** Upper panel: May-Grünwald-Giemsa staining of bone marrow aspirate smears at a ×100 magnification. Lower panel: Sanger sequencing of bone marrow AML cells focusing on the region surrounding the c35G>C substitution. **G** Left panel: evolution of the white blood cell count (WBC) and monocyte count dependent on time. Therapeutic interventions are indicated. Right panel: bone marrow sample cultured ex vivo with vehicle or 25 nM trametinib for 24 h. Vertical bars indicate standard deviations. *p < 0.05.

We used a 592 compounds target selective inhibitor library mostly comprising kinase inhibitors, and observed that NF1^KO^ cells were more sensitive to different MEK inhibitors compared to control cells (CTR*, cultured with GM-CSF), while these cells were equally sensitive to the majority of the compounds including p38 inhibitors (Fig. 3A and Supplementary Fig. 3A). These results identify MEK inhibitors as potential agents selectively targeting NF1^KO^ cells, in agreement with the antileukemic activity of MEK inhibitors observed in *Nras*^*G12D*^-driven mouse leukemia models [23]. To further assess this hypothesis we employed the MEK inhibitor trametinib (not part of the library) currently under development for multiple clinical applications in oncology including in AML [25, 26].

Dose-range experiments with trametinib performed in RAS-activated TF-1 and Ba/F3 cells showed that RAS activation status correlated to increased trametinib cytotoxicity, which was relieved by the addition of cytokines (Fig. 3B and Supplementary Fig. 3B, C). Trametinib anti-leukemic activity involved apoptosis induction, as shown by increased flow cytometry annexin V staining, and PARP and caspase 3 cleavage in NF1^KO^ cells (Supplementary Fig. 3C, D). Moreover, trametinib was preferentially active against AML cell lines with RAS activation due to mutations in RAS genes or to the activation of signaling pathways such as FLT3 (Supplementary Fig. 3E, F). We further observed that trametinib significantly prolonged the survival of mice transplanted with NF1^KO^ TF-1 cells (Fig. 3C, left panel). Moreover, trametinib efficiently inhibited ERK phosphorylation in bone marrow leukemic cells from these mice (Fig. 3C, right panel). These models of genetic RAS activation thus demonstrated an addiction to oncogenic RAS−MEK signaling.

We performed leukemia colony-forming unit (L-CFU) assays in 69 primary samples of AML patients from our cohort, among which 37 (53.5%) had a significant colony formation (i.e., formation of at least ten L-CFU) ex vivo. In these samples, RAS status had no impact on the clonogenic potential of AML samples (Supplementary Fig. 3G), and trametinib reduced the absolute number of L-CFU regardless of RAS status compared to the vehicle condition (Fig. 3D). However, L-CFU formation was more efficiently reduced in RAS− compared to RAS+ samples (Fig. 3E, left panel). Among RAS+ samples, *NRAS* G12A/V/S and Q61R mutations had the highest sensitivity to trametinib, while *KRAS* and other mutations (including *PTPN11*, *NF1*, *CBL*, *RAF1*, *SOS1*, and one case of *NRAS* Q61L) were less sensitive and even showed an increased colony formation in four cases (Fig. 3E, right panel). Moreover, ERK phosphorylation was inhibited by trametinib in both RAS− and RAS+ samples, including in some RAS+ samples with low sensitivity to trametinib in L-CFU assays (Supplementary Fig. 3H). From the Hematobio study (ClinicalTrials.gov NCT02320656) assessing ex vivo sensitivity of AML cells to a panel of compounds [27], 107 cases were available for RAS status, and we observed that Trametinib activity was slightly higher in RAS+ compared to RAS− samples in short-term culture assays (Supplementary Fig. 3I). Similar results were observed in the BEAT AML database with two MEK inhibitors Trametinib and Selumetinib (Supplementary Fig. 3J).

We treated with trametinib an 84 years old woman having a *NRAS*^G12A^-mutated monocytic AML in the absence of any other therapeutic options (Fig. 3F). She first received 2000 mg daily hydroxycarbamide, which due to limited efficacy and hematological toxicity was switched for 2 mg/d trametinib after 3 weeks. During 10 days of trametinib therapy, her WBC and monocyte count were at their lowest values. After trametinib discontinuation due to neurological side effects, WBC and monocyte count markedly increased. A second course of trametinib again dramatically reduced leukocytosis, before the definitive discontinuation of this molecule due to neurological toxicity (Fig. 3G, left panel). After incubation of the leukemic cells of the patient with trametinib ex vivo, we observed a complete inhibition of ERK phosphorylation compared to vehicle-treated cells (Fig. 3G, right panel). These results suggested that trametinib exerted an on-target clinical anti-leukemic activity in this patient.

Collectively, these results suggested that ex vivo sensitivity of RAS+ AML samples to MEK inhibitors was heterogeneous and governed by the nature of RAS pathway genes mutations.

### Identification of pyrvinium pamoate as antileukemic compound active in RAS+ AML

**Fig. 4:**
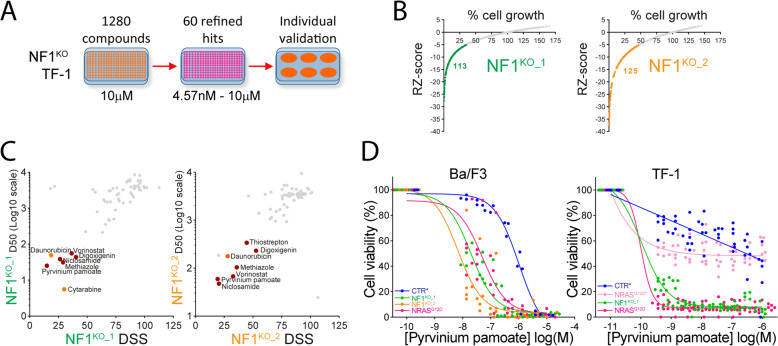
Identification of pyrvinium pamoate as anti-leukemic compound active in RAS+ AML. **A** Schematic representation of high-density pharmacological screen in NF1 KO TF-1 cells. **B** First screen using the PCL library of 1280 compounds at 10μM and CellTiter-Glo® cell viability reagent after 72 h of incubation. Results are represented for each compound (identified by a single dot) by the relation between their robust Z-score value (RZ-score) in Y-axis and the percentage of cell growth in X-axis. Compounds with a RZ-score ≤ −5 (retained for further analysis) are highlighted in red. **C** Second screen performed with serial dilutions of the top-60 compounds from the first screen in NF1 KO TF-1 cells. Results are presented for each compound illustrated by a dot as the correspondence between their median effective dose (ED50, represented using a Log10 scale) and drug sensitivity score (DSS). The best hits are highlighted in red, and the classical AML chemotherapies (daunorubicin and cytarabine) are highlighted in orange. **D** Dose-range experiments using log-dilutions (10^−5^ to 10^−8 ^M) of pyrvinium pamoate in CTR (non-targeting sgRNA), NF1^KO-1^, NF1^KO_2^ and NRAS^G12D^ Ba/F3 cells (left panel) or TF-1 (right panel) cells during 48 h. * indicate culture in the presence of IL-3 (Ba/F3 cells) or GM-CSF (TF-1 cells). Results in the control condition of each compound were normalized to all control conditions across each plate.

We searched for new compounds active in RAS+ AML in a 1280 PCL library (Fig. 4A). Among cell lines demonstrating a similar addiction to oncogenic RAS activation, we retained NF1^KO^ rather than NRAS^G12D^ TF-1 cells to avoid bias due to the overexpression of an ectopic allele. A first screen was performed at 10 µM for each compound, and we selected 113 and 125 compounds inducing significant cell growth inhibition in NF1^KO_1^ and NF1^KO_2^ cell lines, respectively (Fig. 4B and Supplementary Fig. 4A). After refinement of these hits and elimination of redundant compounds in terms of chemical structure and/or pharmacodynamics, the top-60 compounds were selected for dose–response experiments (10^−6^–4.57 × 10^−9 ^M) over the same cell lines. Based on median dose–effect (effective dose 50, ED50) and drug sensitivity score (DSS), we selected 6 compounds as having the strongest cytotoxic activity against NF1^KO^ cells, within the same range than cytarabine and daunorubicin, two key AML chemotherapies (Fig. 4C and Supplementary Fig. 4B). These compounds were Niclosamide, an anthelmintic drug with mitochondria uncoupling properties [28, 29], methiazole (used to treat hyperthyroidism), thiostrepton (a peptidic antimicrobial compound), vorinostat (an HDAC inhibitor used in T-cell lymphoma [30]), digoxigenin (a plant-derived small molecule with antigenicity properties and ability to conjugate to biomolecules), and pyrvinium pamoate, an oral anthelminthic drug employed in pinworm infection [31]. The same experiments were performed in CTR TF-1 cells cultured with GM-CSF, and similar results were observed, while the effect of most of these compounds was more pronounced in NF1^KO^ TF-1 cells (Supplementary Fig. 4C). Indeed, TF-1 cells harbored a *NRAS* G61P mutation (Supplementary Table 1), which together with GM-CSF stimulation may have contributed to RAS activation in these cells. After an individual in vitro validation assay using RAS−activated TF-1 cells, we focused on a quinolone-derived molecule, pyrvinium pamoate, which had the largest differential effect between RAS+ and RAS− cells among the selected compounds (Fig. 4D and Supplementary Fig. 4D).

In activated RAS Ba/F3 cells, a minimal model of oncogene dependency widely employed in drug screening [32], pyrvinium pamoate decreased the viability of IL3-starved NF1^KO^ and *NRAS*^G12D^ cells compared to control cells cultured in the presence of IL-3 (Fig. 4D, left panel and Supplemental Fig. 4E). We then used the isogenic TF-1 cells model, in which oncogenic dependency to RAS−MAPK signaling is relieved by the addition of GM-CSF. In this model, pyrvinium was markedly more cytotoxic against GM-CSF-starved NF1^KO^ or NRAS^G12D^ cells compared to control cells or to NRAS^G12D^ cells cultured with GM-CSF, suggesting that antileukemic activity of pyrvinium occurred in RAS−addicted cells (Fig. 4D, right panel). Together these results showed that pyrvinium pamoate was preferentially cytotoxic to AML cells with mutation-driven RAS activation.

### Pyrvinium pamoate targets mitochondrial respiration in AML

**Fig. 5:**
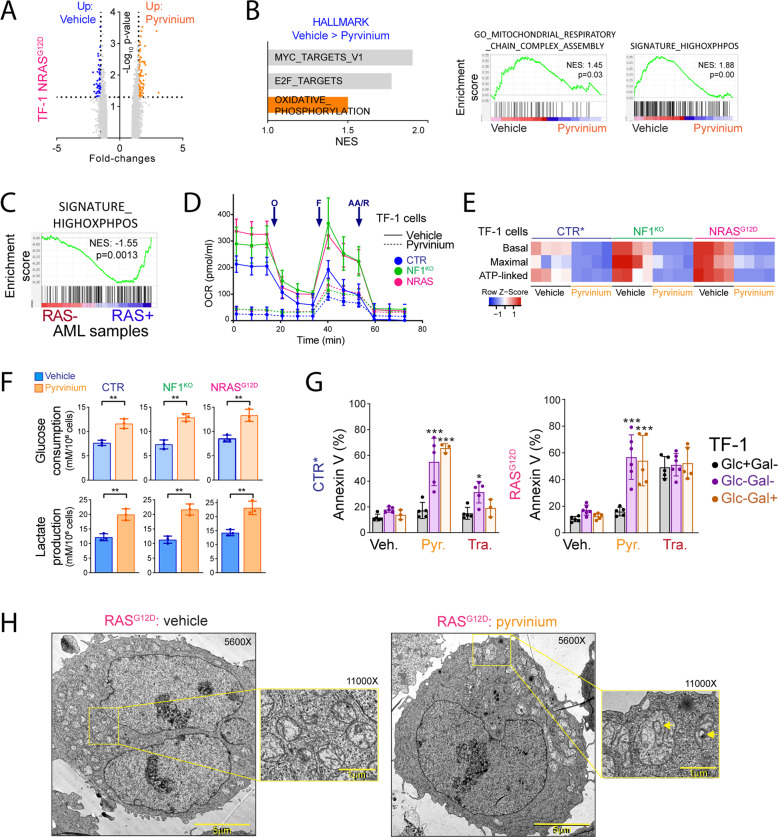
Pyrvinium pamoate targets mitochondrial respiration in AML. **A** Gene expression profiling using microarrays in NRAS^G12D^ TF-1 cells incubated with vehicle or pyrvinium for 6 h. Genes with significant changes (fold-change ≥ 1.5 and *p*-value < 0.05) between the Vehicle and Pyrvinium conditions are highlighted (up in Pyrvinium in orange, up in Vehicle in blue). **B** Left panel: representation of the three significantly enriched Hallmark gene sets in Vehicle compared to Pyrvinium conditions based on their normalized enrichment score (NES). Right panel: representation of two enrichment plots of mitochondrial respiration and oxidative phosphorylation gene sets. **C** Enrichment plot using the high OxPhos signature from the transcriptomic analysis of RAS + versus RAS- AML samples. CTR, NF1^KO_1^ or NRAS^G12D^ TF-1 cells were incubated during 6 h with vehicle (CTR) or 500 nM pyrvinium pamoate in bioenergetic analysis assays measuring the oxygen consumption rate (OCR). **D** OCR evolution dependent on time. O: olygomycin; F: FCCP; AA/R: antimycin A/rotenone. **E** Heatmap representation of calculated basal, maximal and ATP-linked OCR. **F** Glucose consumption and lactate production in CTR, NF1^KO^, and NRAS^G12D^ TF-1 cells incubated with vehicle or pyrvinium for 24 h. **G** Apoptosis measured by flow cytometry annexin V binding assays in CTR (cultured with GM-CSF as indicated by *) or NRAS^G12D^ TF-1 cells incubated with vehicle, 750 nM pyrvinium or 25 nM trametinib in standard (Glc+ Gal+), glucose deficient (Glc-Gal-) or glucose-deficient and galactose-supplemented (Glc-Gal+) culture medium. **H** NRAS^G12D^ TF-1 cells were incubated 6 h with vehicle or 500 nM pyrvinium. Electron microscopy was performed at a 5600× or 11,000× magnification, as indicated. The size scale is provided for each image. Vertical bars indicate standard deviations. *p < 0.05; **p < 0.01; ***p < 0.001.

We investigated the biological activity of pyrvinium pamoate in RAS+ AML models using gene expression profiling performed in NRAS^G12D^ TF-1 cells incubated 6 h with vehicle or pyrvinium pamoate. Differential gene expression was modest between vehicle and pyrvinium conditions as expected at this early time-point (103 and 62 genes up and downregulated, respectively, in pyrvinium-treated cells, Fig. 5A). However, we observed that the oxidative phosphorylation signature was one of the three Hallmark gene sets significantly enriched in vehicle compared to pyrvinium-treated cells (Fig. 5B, left panel). Accordingly, individual analysis revealed a significant enrichment in other mitochondria-related gene sets, including the high OxPhos signature established in AML cells [33] (Fig. 5B, right panel). Interestingly, the same high OxPhos signature was enriched in RAS+ compared to RAS− samples from AML patients, as well as other metabolic signatures (fructose and mannose, pentose phosphate and galactose) suggesting that samples with mutations in RAS pathway genes have increased metaboslim that may represent a vulnerability in AML (Fig. 5C and Supplementary Fig. 5A).

We performed bioenergetics assays and observed that the oxygen consumption rate (OCR) was higher, and that the mitochondrial electron transport chain complex I inhibitor IACS-010759 was preferentially cytotoxic in NF1^KO^ and NRAS^G12D^ compared to CTR TF-1 cells cultured with GM-CSF, suggesting that RAS−activated AML cells were addicted to mitochondrial oxidative metabolism (Fig. 5D and Supplementalry Fig. 5B). Moreover, incubation with pyrvinium near-completely inhibited basal and ATP-linked OCR in all the conditions, while maximal OCR was inhibited in RAS−activated but not in control cells (Fig. 5D, E). In Ba/F3 cells, OCR was higher in control cells compared to NF1^KO^ and NRAS^G12D^ cells incubated with or without IL-3, respectively, possibly due to a direct impact of IL3- receptor signaling on mitochondrial respiration (Supplementary Fig. 5C, D). However, pyrvinium inhibited maximal OCR in RAS−activated but not in control Ba/F3 cells, suggesting that pyrvinium preferentially targeted mitochondrial respiration in RAS−activated cells (Supplementary Fig. 5C, D). We also investigated glycolysis by measuring glucose consumption and lactate production in cell culture supernatants, and observed that treatment with pyrvinium enhanced glycolysis, attested by both increased glucose consumption and lactate production in TF-1 and Ba/F3 cells regardless the introduction of RAS−activating genetic events (Fig. 5F and Supplementary Fig. 5E). In contrast, trametinib had a limited effect on OCR, and moderately decreased glucose consumption and lactate production in TF-1 cells (Supplementary Fig. 5F, G). Taken together, these results suggested that pyrvinium, but not trametinib inhibited mitochondrial respiration, leading to an increased dependency on glucose metabolism for AML cells to survive.

Galactose may sustain cytosolic glycolysis, thus providing precursors to TCA cycle, allowing human cells with functional mitochondria to survive in the absence of glucose [34]. In glucose-starved TF-1 CTR cells, trametinib enhanced apoptosis, and this effect was rescued by culture in the presence of galactose, suggesting that parental TF-1 cells had efficient mitochondria and that trametinib only modestly affected their function (Fig. 5G, left panel). In RAS^G12D^ TF-1 cells, trametinib induced apoptosis even in the presence of glucose, and this effect was not modified by glucose starvation or addition of galactose (Fig. 5G, left panel). In TF-1 CTR or NRAS^G12D^ cells, pyrvinium did not induce annexin V positivity in standard culture conditions after 24 h, while a marked apoptotic response that was not rescued by galactose was observed in glucose-starved cells (Fig. 5G, right panel). These results confirmed that pyrvinium induced a mitochondrial defect in AML cells that was unmasked in the absence of glucose. We further performed electron microscopy experiments in NRAS^G12D^ TF-1 cells cultured 6 h with vehicle or pyrvinium, and observed heterogeneously distributed mitochondria of altered morphology including disorganized cristae and swollen membranes as indicated by arrows in pyrvinium-treated compared to vehicle-treated TF-1 cells (Fig. 5H), suggesting that pyrvinium efficiently targeted mitochondria in AML cells. Collectively, these results indicated that inhibition of mitochondrial respiration participated in the antileukemic effects of pyrvinium.

### Synergy between the MEK inhibitor trametinib and pyrvinium pamoate in RAS activated cells

**Fig. 6:**
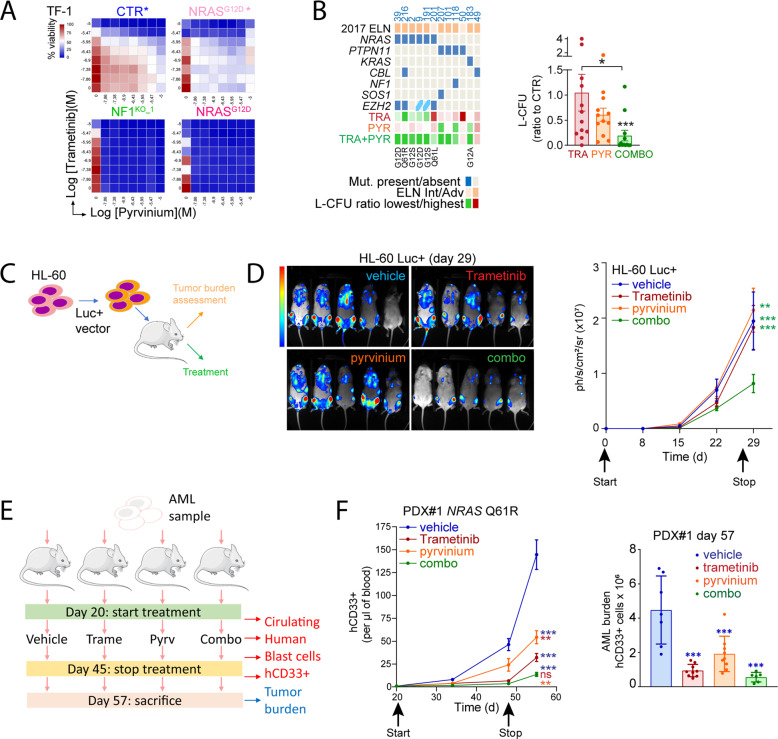
Synergy between the MEK inhibitor trametinib and pyrvinium pamoate in RAS activated cells. **A** Combination drugs dose-range assays in CTR (with GM-CSF as indicated by *), NF1 KO and NRAS^G12D^ (without or with GM-CSF) TF-1 cells incubated with pyrvinium and/or trametinib for 48 h. Heat maps provided the combined results of three independent experiments. **B** L-CFU assays in RAS + primary AML samples incubated with vehicle, 50 nM trametinib, 250 nM pyrvinium pamoate or trametinib/pyrvinium combination during 7 days. Left panel: individual data for ELN risk category and *NRAS*, *PTPN11*, *KRAS*, *CBL*, and *SOS1* mutations on the 12 samples. Type of amino acid substitutions are provided for *NRAS* and *KRAS* mutations. Right panel: pool of the individual values on L-CFU (presented as a ratio between L-CFU number in each condition relative to the vehicle-treated condition). In vivo luminescence assays using the HL-60 cell line. **C** Experimental plan: the HL-60 cell line transduced with a vector expressing luciferase (HL-60 Luc+) was injected into immunodeficient NSG recipient mice. Treatment with vehicle, 0.25 mg/kg/d trametinib (oral gavage, OG), 0.5 mg/kg pyrvinium (intraperitoneal injection, IP) or combination of trametinib and pyrvinium started the day of transplantation (*N* = 5 mice per treatment group). Tumor burden was measured using a luminescent camera every week. **D** Left panel: images captured at day 29 from treatment onset in the four experimental groups. Right panel: quantification of the luminescent signal representing HL-60 Luc+ tumor burden from treatment onset during 4 weeks. Patient-derived xenograft (PDX) experiment in immunodeficient NSG mice recipients. **E** Experimental plan: AML cells were propagated to nine mice per experimental group (vehicle, trametinib, pyrvinium and combination) and treatment started 20 days after transplantation when human AML cells were detected in mice peripheral blood. Treatment with vehicle, 0.25 mg/kg/d trametinib (OG), 0.25 mg/kg/d pyrvinium (IP) or combination of trametinib and pyrvinium was given by daily oral gavage during 25 days and then discontinued. **F** Left panel: disease propagation was monitored every 2 weeks by flow cytometry with antihuman (h)CD33 antibody in mice peripheral blood. Right panel: tumor burden was assessed at the end of the experiment on total bone marrow and spleen using anti-hCD33 antibody. Vertical bars indicate standard deviations. ***p < 0.001.

Despite the oncogenic addiction induced by genetic alterations of RAS pathway genes in AML, the clinical activity of MEK inhibitors appeared limited in RAS+ AML [35]. The inhibition of mitochondrial respiration by pyrvinium, but not by trametinib suggested distinct pharmacodynamics profiles and potential benefits of their association against AML cells. Single-agent pyrvinium or trametinib were more active in GM-CSF-starved NF1^KO^ and NRAS^G12D^ TF-1 cells compared to control or NRAS^G12D^ TF-1 cells cultured with GM-CSF, and similar results were observed in Ba/F3 cells (Fig. 6A and Supplemental Fig. 6A). As pyrvinium was markedly cytotoxic to NF1^KO^ and NRAS^G12D^ TF-1 cells, synergy with trametinib could not be assessed, but synergy was observed in NF1^KO^ and NRAS^G12D^ Ba/F3 cells but not in control cells (Supplementary Fig. 6A).

Compared to short-term liquid culture assays, L-CFU assays investigate immature AML progenitor cell proliferation and survival capacities over a 7-10 days period [36]. We performed L-CFU assays in 12 RAS+ primary AML samples incubated with vehicle, trametinib, pyrvinium or trametinib, and pyrvinium combination (Fig. 6B). Inhibition of AML cells clonogenic capacities was heterogeneous in the presence of trametinib or pyrvinium single-agents, but interestingly samples with resistance to one compound were generally sensitive to the other, and more sensitive to the combination (Fig. 6B and Supplemental Fig. 6B). In pooled analysis, neither trametinib nor pyrvinium significantly reduced colonies formation, possibly reflecting their heterogeneous activity particularly dependent on the genetic background of each sample, but combination of these two molecules significantly reduced L-CFU compared to the control and trametinib conditions (Fig. 6B, right panel).

To investigate the preclinical potential of the trametinib and pyrvinium association, we first transplanted the luciferase-expressing (Luc+) RAS+ HL-60 AML cell line to immunodeficient NOD-SCID gamma-null (NSG) mice (Fig. 6C). We followed-up the evolution of tumor burden every week by direct luminescence quantification, and observed that treatment with trametinib and pyrvinium association significantly reduced tumor burden compared to single-agents or placebo (Fig. 6D). Moreover, pyrvinium and trametinib combination slightly improved survival compared to each single agent in this aggressive AML mouse model (Supplementary Fig. 6C). During this experiment, we did not detect a significant toxicity of pyrvinium single agent or combined to trametinib, as attested by mice weight evolution and hematopoietic cells quantification (Supplementary Fig. 6D, E). We then performed a patient-derived xenograft (PDX) experiment with a sample harboring a *NRAS* Q61R mutation injected to nine mice per treatment group as vehicle, trametinib, pyrvinium and combination. Treatments started 20 days after injection after the detection of human AML cells in mice blood and were given during 25 days, and disease propagation was evaluated by human CD33-positive (hCD33+) leukemic cells flow cytometry detection in mice blood until total tumor burden measure after sacrifice at day 57 (Fig. 6E). We observed that pyrvinium, trametinib, and combination significantly reduced circulating hCD33+ cells and AML tumor burden compared to vehicle, and a trend to enhanced activity of combination, even if single-agents were significantly active in this case (Fig. 6F). While three mice died early after treatment onset in the combination group, subsequent decrease of pyrvinium dose from 0.5 to 0.25 mg/kg/d allowed the completion of the experiment without toxicity, including on mice hematopoietic system (Supplementary Fig. 6F, G). Collectively, these results indicated that pyrvinium and trametinib, targeting distinct cellular processes, had synergistic anti-leukemic activity in vitro against AML cell lines and primary samples from patients, and that their association efficiently inhibited the propagation of human AML in vivo in mice.

## Discussion

RAS was the first oncogene identified in human cancers, and its implication in oncogenesis has been widely studied since [2]. While the resolution of AML genetic landscape allowed the identification of molecular subgroups of patients with prognostic and/or therapeutic significance [37], RAS pathway mutations were generally not considered to define a specific entity. Molecular mechanisms regulating the balance between activated RAS−GTP and inactive RAS−GDP are complex, involving multiple effectors such as protein kinases, scaffolding proteins, phosphatases, GAPs, and GEFs [38]. Mutations in genes encoding actors of this complex network are found in inherited genetic syndromes referred to as RASopathies genes and in sporadic cancers, including in AML and at a high frequency in juvenile myelomonocytic leukemia (JMML) [19]. We investigated the potential impact of mutations in these RASopathies genes (RAS+) in AML from descriptive, prognostic, and preclinical therapeutic perspectives.

To focus on AML with unmet prognostic and therapeutic needs, we excluded from our cohort AML cases of the favorable ELN prognostic group (*t*(8;21), inv(16), bi-allelic *CEBPA*, and *NPM1* without *FLT3-ITD* mutations) [7]. We also excluded *FLT3-ITD* cases that generally do not harbor RAS pathway mutations at diagnosis (Supplementary Fig. 1B). Small RAS−mutated subclones may preexist in FLT3-ITD samples, as RAS mutations are frequently detected in samples from patients after failure of tyrosine kinase inhibitors [18]. However, we did not detect RAS pathway mutations in six FLT3-ITD samples using our NGS panel (Supplementary Fig. 1I), suggesting that these subclones may fall outside of the detection threshold of conventional sequencing techniques.

From the 127 patients of our cohort, we identified at least one RAS pathway alteration in 40% of AML cases. The proportion of RAS mutations range between 25% and 40% in large AML genomic studies and may depend on the depth of sequencing technique employed allowing the detection of small-sized subclones [21, 37, 39, 40]. Besides using deep targeted sequencing at an average depth of 875 reads and a 1% VAF cutoff in our RAS sequencing panel, exclusion from our analysis of favorable ELN and FLT3-ITD cases may also account for the high rate of RAS mutations detection in our study. The most prevalent alterations concerned *NF1*, *NRAS, KRAS,* and *PTPN11*, but we also observed variants affecting *CBL, BRAF, RASA1, SOS1, MAP2K2*, and *RAF1* genes. We did not detect mutation in RAS pathways negative regulators, including *DUSP6*, *SPRED,* and *SPRY* family members. However, we identified an increased frequency of PRC2 alterations in RAS+ samples, as reported in JMML [41, 42] and other cancer types [10, 43], which may amplify oncogenic RAS signaling in AML. The distribution of myeloid-related gene mutations was similar between RAS+ and RAS− cases, except for *IDH2* mutations more frequently detected in RAS− samples, as reported [37].

From RNAseq in 52 samples from the cohort, we did not detect a clear clustering of RAS+ samples, as reported [20], suggesting that the impact of RAS activation on gene expression was low with respect to other molecular drivers in AML. However, we observed an enrichment in RAS−related genes and signatures in RAS+ samples, in agreement with increased ERK phosphorylation detected in RAS+ samples. These results highlighted the biological consequences of RAS mutations in AML.

We investigated the prognostic impact of RAS pathway alterations in 79 AML patients from our cohort homogeneously treated with intensive anthracycline- and cytarabine-based induction regimens. Recent studies found frequent *NRAS* and *KRAS* mutations in the ELN favorable core-binding factors AML subtype, and showed that the presence of subclones carrying RAS gene mutations identified, among these seemingly good prognostic patients, those having a reduced survival probability [44]. While *NRAS* and/or *KRAS* mutations were reported as having a neutral impact on survival in AML [45, 46], a recent study on a large cohort of intensively-treated AML patients over 60 showed that *NRAS* or *KRAS* mutations had an independent negative impact on survival [47]. Moreover, *NF1* mutations were associated with reduced survival probability among the adverse cytogenetic subgroup of AML [48, 49]. In our cohort, we observed that only *KRAS* mutations had prognostic significance when considered individually, but that the RAS+ group aggregating RAS pathway mutation cases had a reduced survival probability, particularly within the ELN intermediate prognostic group. From our analysis of the BEAT AML database using the same filters as in our study, we also observed a near-significant reduced survival probability in RAS+ ELN intermediate AML patients, even if those mutations were reported in only 19.5% of this category, as compared to 32.1% in our cohort. Interestingly, RAS mutations were significantly associated with increased cell proliferation markers including elevated white blood cell count, blast cell percentage, and LDH levels. Our data suggested that the presence of RAS activating mutations might define a group of patients having a similar clinical course upon intensive induction chemotherapy.

We took advantage of growth-factor-dependent cell lines to model the most frequent RAS pathways alterations detected in AML patients. We deleted *NF1* by CRISPR/Cas9, and expressed *NRAS* G12D or *PTPN11* D61Y mutant forms in these cells, which grew in culture independent of cytokines, as reported [50]. We confirmed RAS pathway activation in these cells, as well as increased oncogenic potential when transplanted to immunodeficient mice in the case of *NF1* KO cells. Moreover, these RAS−activated cells demonstrated an increased sensitivity to the MEK inhibitor trametinib in vitro, and in vivo in *NF1* KO cells, indicating that these cell line models became addicted to oncogenic RAS signaling [6].

However, the antileukemic activity of trametinib appeared heterogeneous in primary samples from AML patients when assayed ex vivo in colony formation assays, with a significantly lower efficacy against *KRAS*- or *PTPN11*-mutated compared to *NRAS* or non-RAS−mutated samples, as reported [6]. In contrast, we observed a superior activity of trametinib against RAS+ compared to RAS− samples among 114 primary AML samples assayed in short-term liquid cultures assays, which was corroborated by our analysis of the BEAT AML data on the activity of trametinib and selumetinib. We could hypothesize that the differential effect of trametinib between L-CFU and short-term liquid culture reflected the different cell populations assayed. Indeed, L-CFU assays enrich for more immature AML progenitor cells compared to liquid culture conditions [51], suggesting a reduced sensitivity to MEK inhibitors of progenitor compared to mature RAS+ cells. Future studies should investigate these potential intrinsic barriers to MEK inhibitor activity using in vivo RAS+ AML models. While we observed a transient on-target activity of trametinib in a *NRAS* G12D-mutated AML patient, illustrating the dependency of AML cells to RAS mutations, results of early phases MEK inhibitors clinical trials found a limited efficacy of these molecules [25, 35, 52]. Collectively, these preclinical and clinical data suggested a need for combination strategies with MEK inhibitors to efficiently treat RAS−dependent cancers [6].

To repurpose molecules potentially targeting RAS−activated cells, we screened *NF1* KO AML cells using a large FDA-approved compound library and identified pyrvinium pamoate, an oral anthelminthic drug employed in pinworm infection [31]. Other interesting molecules were identified during this screen such as niclosamide, but some of them appeared less cytotoxic in additional validation assays and/or did not showed a superior activity in RAS+ compared to RAS− cells. In contrast, mutation-induced RAS activation Ba/F3 and TF-1 cells were markedly more sensitive to pyrvinium compared to parental cells. Moreover, as previously reported in CRISPR/Cas9-based gene essentiality profiling experiments [4], this effect was relieved by the addition of GM-CSF or IL-3 in TF-1 or Ba/F3 cell lines, respectively, suggesting that RAS activation determined a new vulnerability to this compound. While mutation-driven RAS activation governed cellular response to pyrvinium in minimal cellular models, the effect of pyrvinium—or to trametinib—was not selective for RAS mutated AML samples, but dependent on RAS signaling pathway activation, as illustrated by the sensitivity of FLT3-mutated AML to MEK inhibitors or to pyrvinium [53, 54]. The anti-cancer activity of pyrvinium recently emerged in several models as targeting several intracellular processes including mitochondrial oxidative metabolism, and signaling such as Wnt and STAT3 signaling pathways [54–57]. Particularly, antileukemic activity of pyrvinium was observed in pediatric MLL-rearranged AML, B-cell acute lymphoblastic leukemia (ALL), and T-ALL with NOTCH inactivation signature [58–60]. Mechanistically, we determined that pyrvinium predominantly inhibited mitochondrial oxydative phosphorylation in RAS+ AML, as observed in other cancers and in *FLT3*-mutated AML [54, 61–63], and this mitochondrial dependency is further illustrated by the anti-leukemic activity of the mitochondria uncoupling agent niclosamide in RAS+ AML cells, as recently demonstrated in T-ALL [64]. Beside complex I mitochondrial respiratory chain inhibition, other mechanisms may account for cytotoxic activity of pyrvinium in cancer, including inhibition of STAT3 phosphorylation possibly mediated by AMP-activated protein kinase, induction of an endoplasmic reticulum stress response, and inhibition of Wnt signaling [67]. In contrast, the MEK inhibitor trametinib had a limited impact on mitochondrial respiration and decreased glucose consumption and lactate production as reported in other models [65, 66].

The complementary metabolic profile of trametinib and pyrvinium in AML cells led to combination experiments, and we observed synergy between these two compounds in vitro in RAS−activated cell lines models. In primary samples from AML patients, pyrvinium appeared more active in *PTPN11* and *KRAS* than in *NRAS* mutants, while the opposite was seen for trametinib and interestingly, all but one sample were more sensitive to the combination of the two compounds compared to single-agents. These results suggested that vulnerability or resistance to pyrvinium or trametinib could correlate with RAS mutational status in AML, while combination therapy may overcome these limitations. The recent development of mutation-specific *KRAS* G12C inhibitors highlighted the heterogeneous structural and functional consequences of specific *NRAS* and *KRAS* mutations [5, 68] and we may hypothesize that these different mutations may confer specific metabolic vulnerabilities to AML cells accounting for their sensitivity to trametinib or pyrvinium. Accordingly, the pyrvinium and trametinib association efficiently reduced the propagation of the HL-60 cell line causing a rapidly aggressive disease when transplanted to immunodeficient mice. Moreover, while both trametinib and pyrvinium inhibited the propagation of a RAS+ PDX, combined therapy also appeared more efficient in this model without evidence for increased toxicity. The combination of pyrvinium with a MEK inhibitor thus appeared as a promising personalized therapeutic strategy in RAS+ AML.

Direct pharmacological targeting of activated RAS remains one of the most challenging problem of cancer drug discovery [2]. Targeting signaling pathways or metabolic reprogramming downstream RAS activation thus represents promising alternative strategies. A significant subgroup of AML patients characterized by alterations in RAS activating genes had an adverse outcome on conventional AML therapies. Repurposing of pyrvinium pamoate and/or development of a bioavailable derivative compound to be combined with a MEK inhibitor represent a meaningful therapeutic opportunity for these patients to be investigated in clinical trials.

## Supplementary information

Supplemental Figures and Methods
