# Supplementary material for: RAS activation induces synthetic lethality of MEK inhibition with mitochondrial oxidative metabolism in acute myeloid leukemia
Source: Leukemia. 2022 Mar 30;36(5):1237–52. doi: 10.1038/s41375-022-01541-0 (PMC9061298; doi:10.1038/s41375-022-01541-0)
Supplement: Supplementary file 1 — Supplemental Figures and Methods [file 41375_2022_1541_MOESM1_ESM.pdf]

RAS activation induces synthetic lethality of MEK inhibition with mitochondrial oxidative metabolism in acute myeloid leukemia

Justine Decroocq[1,2], Rudy Birsen[1,2], Camille Montersino[3], Prasad Chaskar[4], Jordi Mano[1,2], Laury Poulain[4], Chloe Friedrich[1,5], Anne-Sophie Alary[1,5], Helene Guermouche[1,5], Ambrine Sahal[6], Guillemette Fouquet[1], Mathilde Gotanègre[6], Federico Simonetta[4], Sarah Mouche[4], Pierre Gestraud[7], Auriane Lescure[8], Elaine Del Nery[8], Claudie Bosc[6], Adrien Grenier[1,2], Fetta Mazed[1,2], Johanna Mondesir[1,2], Nicolas Chapuis[1,5], Liza Ho[9], Aicha Boughalem[10], Marc Lelorc'h[10], Camille Gobeaux[1], Michaela Fontenay[1,5], Christian Recher[5], Norbert Vey[11], Arnaud Guillé[12], Daniel Birnbaum[12], Olivier Hermine[1,13,14], Isabelle Radford-Weiss[10], Petros Tsantoulis[4], Yves Collette[3], Rémy Castellano[3], Jean-Emmanuel Sarry[5], Eric Pasmant[1], Didier Bouscary[1,2], Olivier Kosmider[1,5]and Jerome Tamburini[1,2,4, *]

[1] Université de Paris, Institut Cochin, CNRS UMR8104, INSERM U1016, F-75014 Paris, France

[2] Equipe Labellisée Ligue Nationale Contre le Cancer (LNCC), Paris, France

[3] Centre de Recherche en Cancérologie de Marseille (Cancer Research Center of Marseille.), CRCM, Inserm UMR1068, CNRS UMR7258, Aix Marseille Université U105, Institut Paoli Calmettes, Marseille, France

[4] Translational Research Centre in Onco-hematology, Faculty of Medicine, University of Geneva, 1211, Geneva 4, Switzerland.

[5] Hematology Laboratory, Assistance Publique-Hôpitaux de Paris. Centre-Université de Paris, Cochin Hospital, Paris, France

[6] Cancer Research Center of Toulouse, Unité Mixtes de Recherche 1037 INSERM, Toulouse, France

[7] Bioinformatics Platform- U900, Institut Curie, PSL Research University, Paris, France.

[8] BioPhenics High-Content Screening Laboratory, Cell and Tissue Imaging Facility (PICT-IBiSA), Institut Curie, PSL Research University, Translational Research Department, Paris, France.

[9] Pathology department, Geneva University Hospital, 1211, Geneva 4, Switzerland

[10] Cytogenetic Laboratory, Necker Hospital, Paris, France

[11] Hematology Department, Institut Paoli-Calmettes, Aix-Marseille Université, Marseille, France

[12] Inserm, CNRS, Institut Paoli-Calmettes, CRCM, Predictive Oncology, Aix-Marseille Université, Marseille, France

[13] Service d'Hématologie Adultes, Hôpital Universitaire Necker-Enfants Malades, Assistance Publique Hôpitaux de Paris, France.

[14] Institut Imagine, INSERM U1163, 75015 Paris, France.

[*] To whom correspondence should be adressed : current adress : Geneva University Medical Center, rue Jean Servet 1, 1205 Geneva, Switzerand, email : jerome.tamburinibonnefoy@unige.ch

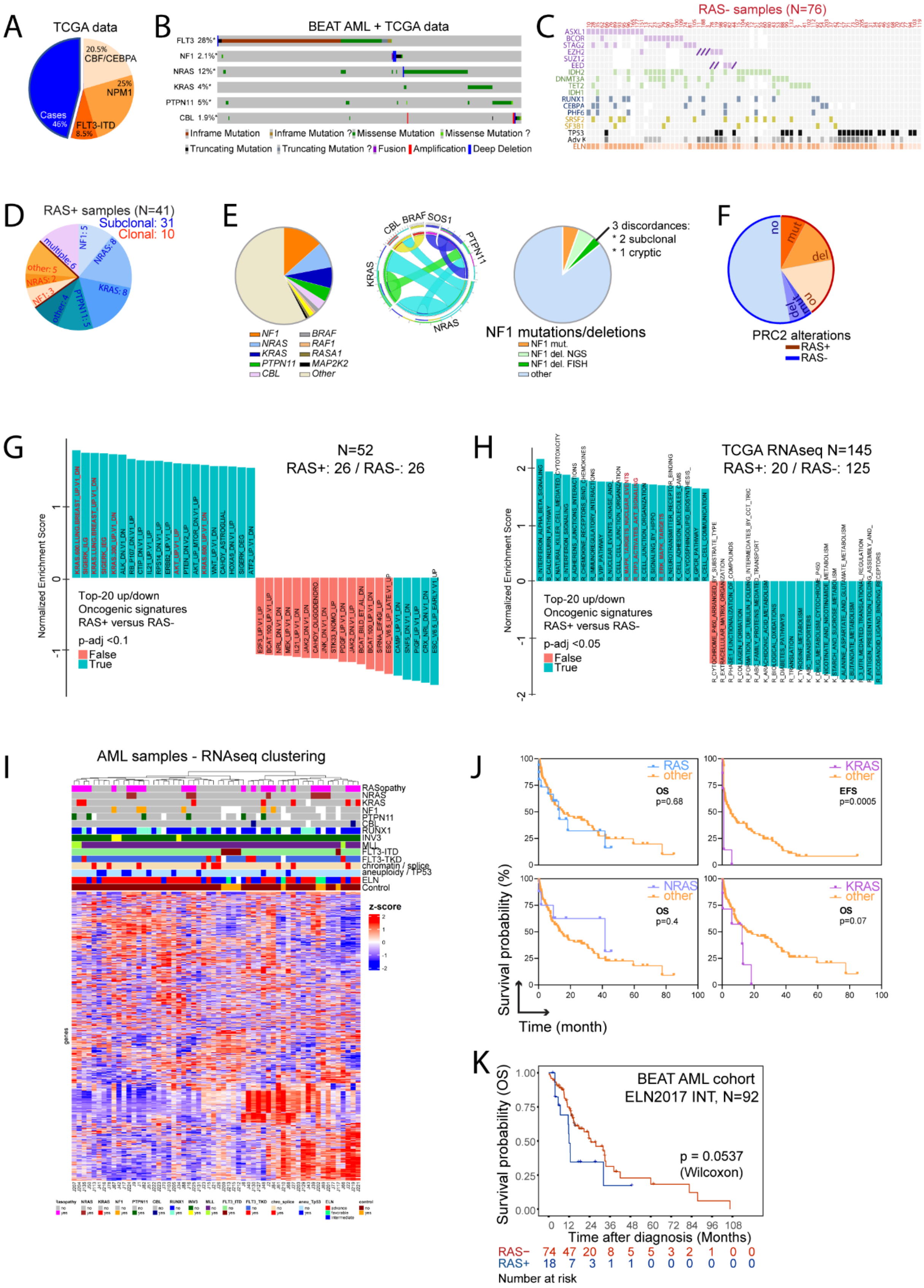

A
TCGA data
20.5% CBF/CEBPA
25% NPM1
Cases 46%
FLT3-ITD 8.5%
B
BEAT AML + TCGA data
FLT3 28%*
NF1 2.1%*
NRAS 12%*
KRAS 4%*
PTPN11 5%*
CBL 1.9%*
Inframe Mutation
Inframe Mutation ?
Missense Mutation
Missense Mutation ?
Truncating Mutation
Truncating Mutation ?
Fusion
Amplification
Deep Deletion
C
RAS- samples (N=76)
ASXL1
BCOR
STAG2
EZH2
SUZ12
EED
IDH2
DNMT3A
TET2
IDH1
RUNX1
CEBPA
PHF6
SRSF2
SF3B1
TP53
ELN
D
RAS+ samples (N=41)
Subclonal: 31
Clonal: 10
NF1: 5
NRAS: 8
KRAS: 8
PTPN11: 5
other: 4
NF1: 3
NRAS: 2
other: 5
multiple: 6
E
NF1
NRAS
KRAS
PTPN11
CBL
BRAF
RAF1
RASA1
MAP2K2
Other
CBL
BRAF
SOS1
PTPN11
KRAS
NRAS
3 discordances:
* 2 subclonal
* 1 cryptic
NF1 mutations/deletions
NF1 mut.
NF1 del. NGS
NF1 del. FISH
other
F
no
mut
del
no
mut
del
PRC2 alterations
RAS+
RAS-
G
N=52
RAS+: 26 / RAS-: 26
Normalized Enrichment Score
Top-20 up/down
Oncogenic signatures
RAS+ versus RAS-
p-adj <0.1
False
True
H
TCGA RNAseq N=145
RAS+: 20 / RAS-: 125
Normalized Enrichment Score
Top-20 up/down
Oncogenic signatures
RAS+ versus RAS-
p-adj <0.05
False
True
I
AML samples - RNAseq clustering
RASopathy
NRAS
KRAS
NF1
PTPN11
CBL
RUNX1
INV3
MLL
FLT3-ITD
FLT3-TKD
chromatin / splice
aneuploidy / TP53
ELN
Control
z-score
genes
J
RAS
other
OS
p=0.68
KRAS
other
EFS
p=0.0005
NRAS
other
OS
p=0.4
KRAS
other
OS
p=0.07
Survival probability (%)
Time (month)
K
BEAT AML cohort
ELN2017 INT, N=92
p = 0.0537
(Wilcoxon)
Survival probability (OS)
Time after diagnosis (Months)
RAS− 74 47 20 8 5 5 3 2 1 0 0
RAS+ 18 7 3 1 1 0 0 0 0 0 0
Number at risk

Supplemental Figure 1. RAS pathway alterations landscape and clinical correlations in AML. A. Repartition of AML cases from the TCGA database dependent CBF fusions (*RUNX1*/*RUNX1T1* and *MYH11*/*CBFB*) and *CEBPA* bi-allelic, *NPM1* and FLT3-ITD mutations detection. Cases (in blue) referred to patients without the aforementioned abnormalities. **B.** Heatmap representing the repartition of *FLT3, NF1, NRAS, KRAS, PTPN11* and *CBL* mutations in the BEAT AML and TCGA cohorts, generated using the cBioPortal online tool. **C.** Next-generation sequencing (NGS) in 76 RAS- diagnosis samples of AML patients. Myeloid neoplasm-related mutations when occurring in at least five samples, regardless the number of mutations detected for a given gene. Variants are indicated by colored squares and deletions by an inclined line. Molecular categories are represented by a color code as indicated in the legend. Cytogenetics (complex and/or adverse karyotype versus others) and European leukemia network 2017 (ELN) risk category (intermediate or adverse) are indicated. **D.** Extrapolation of the clonality of RAS+ mutations based on variant allele frequencies (VAFs) in 41 RAS+ AML samples. **E.** Left panel: distribution of RAS pathway gene mutations across our cohort. Each patient is represented once and only the most frequently detected variant is represented in case of co-mutations. Middle panel: circus plot (computed online using http://mkweb.bcgsc.ca/tableviewer/) representing the nine cases with RAS pathway co-mutations. Right panel: repartition of *NF1* mutations (mut.) and/or deletions (del.). *NF1* deletions detected by fluorescent in situ hybridization (FISH) but not by NGS or karyotype are indicated in dark green as NF1 del. FISH. **F.** Repartition of PRC2 members mutations or deletions (*EZH2, SUZ12* and *EED*) in RAS+ versus RAS- cases. **G.** Transcriptomic analysis of RAS+ versus RAS- AML samples. An enrichment analysis was performed using the oncogenic signature data set, and the top-20 up and down gene sets based on their normalized enrichment score are represented. The true or false discovery correspond to gene sets with an adjusted p-value (p-adj) <0.1 or >0.1, respectively. **H.** An enrichment analysis between RAS+ and RAS- samples using the oncogenic signature data set was performed on RNAseq data (N=145 AML cases) from the TCGA, and the top-20 up and down gene sets based on their normalized enrichment score are represented. The true or false discovery correspond to gene sets with an adjusted p-value (p-adj) <0.1 or >0.1, respectively. **I.** Hierarchical clustering from transcriptomic analysis of 66 primary AML samples. Several characteristic of AML patient samples were investigated including RASopathy (ie. presence of a RAS-activating mutation); *NRAS*, *KRAS*, *PTPN11*, *NF1* or *CBL* mutations; RUNX1: presence of a *RUNX1* mutation; INV3*: presence of an inv(3) chromosomal abnormality involving EVI1, MLL*: presence of a *KMT2A* fusion gene; chromatin / splice*: presence of mutations of the chromatin/splice category; aneuploidy / TP53*: presence of mutations of the aneuploidy/*TP53* category; ELN: European Leukemia Network 0, 1 or 2 score; control: panel of AML samples with different genetic characteristics compared to our target population (ie. *FLT3* mutation, CBF fusion gene…). *: AML molecular subgroups according to [1]. **J.** Analysis of event-free and overall survival (EFS and OS, respectively) dependent on the *NRAS* or/and *KRAS* mutation status in a cohort of 79 AML patients treated with intensive chemotherapy. **K.** Overall survival (OS) in 92 ELN INT AML patients from the BEAT AML cohort having received induction chemotherapy.

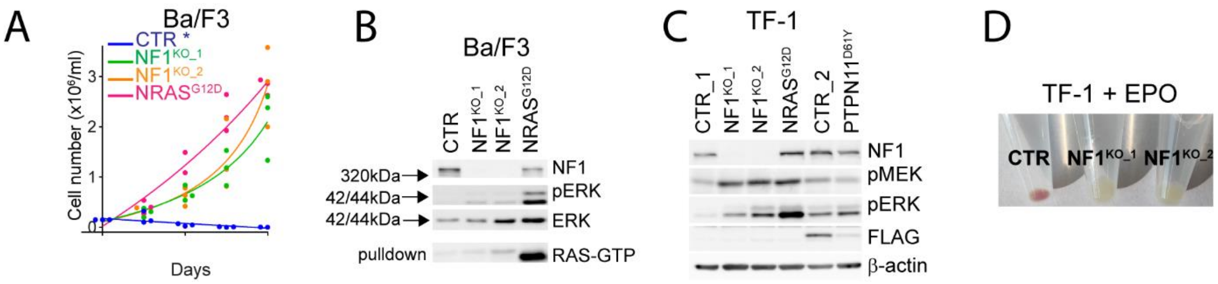

Supplemental Figure 2. Modelization of RAS pathway gene alterations revealed oncogenic addiction in AML. A. Cell counting using trypan blue exclusion assay in CTR (with IL3), NF1^{KO_1}, NF1^{KO_2} and NRAS^{G12D} Ba/F3 cells. **B.** Immunoblotting of total cell lysates and of RAS-RAF1 pulldowns from CTR, NF1^{KO_1}, NF1^{KO_2} and NRAS^{G12D} Ba/F3 cells with antibodies directed against NF1, phospho-ERK, ERK and RAS. Black arrows indicate molecular weight of each proteins. **C.** Immunoblotting of total cell lysates from CTR_1, NF1^{KO_1}, NF1^{KO_2}, NRAS^{G12D}, CTR_2 and PTPN11^{D61Y} TF-1 cells using anti-NF1, -phospho-MEK and -phospho-ERK. PTPN11 ectopic expression was detected by anti-FLAG antibody. **D.** CTR, NF1^{KO_1} and NF1^{KO_2} TF-1 cells were cultured with EPO for7 days. Cell were spun down and hemoglobinization was assessed by red coloration of the pellets.

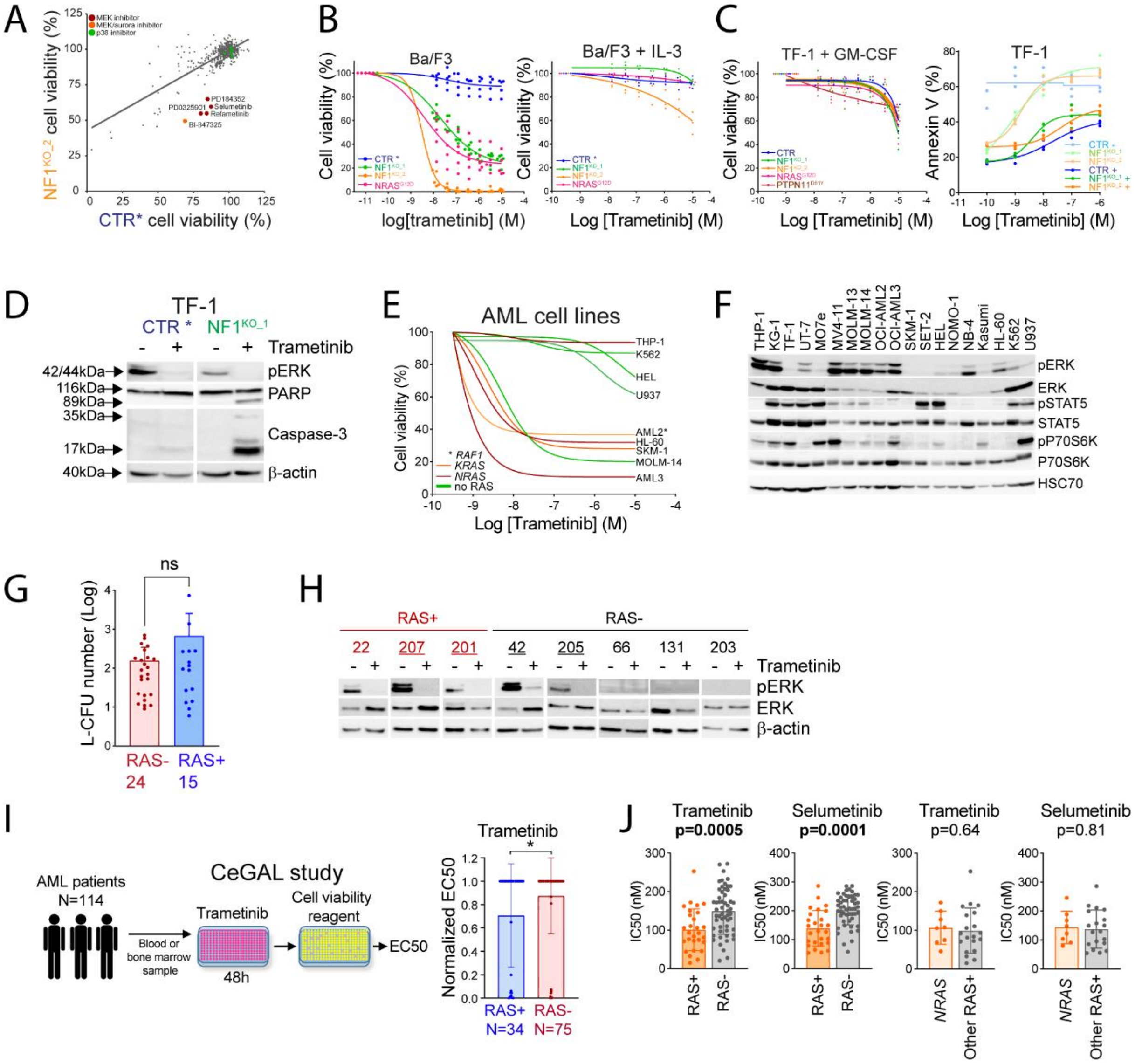

Supplemental Figure 3. Heterogeneous activity of MEK inhibitors against RAS+ AML. A. We applied the target selective inhibitors library (592 unique compounds) to CTR (cultured with GM-CSF as indicated by *) and NF1^{KO_2} TF-1 cells for 48h, before cell viability quantification using the uptiblue fluorescent reagent. Each dot on the graph represents the mean of three independent experiments. **B.** Dose-response curves of the MEK inhibitor trametinib (from 10^{-6} to 13.7×10^{-9}M) on CTR (with IL-3, as indicated by *), NF1^{KO_1}, NF1^{KO_2} and NRAS^{G12D} Ba/F3 cells. The uptiblue luminescent reagent measured cell viability. **C.** Experiments in CTR, NF1^{KO_1} and NF1^{KO_2} TF-1 and Ba/F3 cells cultured with GM-CSF (TF-1) or IL-3 (Ba/F3) as indicated and dose-range concentrations of trametinib. Left panel: cell viability measured by the uptiblue reagent in TF-1 cells cultured with GM-CSF. Middle panel: annexin V flow cytometry assay in TF-1 cells cultured without or with GM-CSF as indicated. Right panel: cell viability measured by the uptiblue reagent in Ba/F3 cells cultured with IL-3. **D.** CTR (with GM-CSF, as indicated by *) and NF1^{KO_1} TF-1 cells were cultured with or without 5nM trametinib for 24h. Protein extracts were immunoblotted using anti-phospho-ERK, anti-PARP, anti-caspase-3 and anti-β-actin antibodies. **E.** Nine human AML cell lines with different RAS mutational status were assayed for in vitro sensitivity to trametinib. The OCI-AML2 cell line was recently shown to bear a *RAF1* activating fusion [2]. We detected a *KRAS*-mutated subclone at a 7% VAF in Kasumi-1 cell line, which we did not led us to consider Kasumi-1 among RAS-mutated AML cell lines due to the low VAF and the absence of other reports of this mutation. **F.** Immunoblots in a panel of AML cell lines using anti-phospho-ERK, -phospho-STAT5, -phospho-P70S6K, -ERK, STAT5, P70S6K and HSC70 antibodies. **G.** Number of L-CFU colonies in RAS+ (n=15) and RAS-(n=24) untreated primary AML samples (Log$_{10}$ scale). **H.** Protein

extracts from three and five RAS+ and RAS- primary AML samples, respectively, incubated with vehicle or 25nM trametinib were investigated for ERK phosphorylation and expression by Western blot. Underlined samples have results from L-CFU assays as presented in Figure 3G when the other were not considered as having a significant *ex vivo* L-CFU growth. **I.** In the CeGAL study, 114 AML samples (RAS+, N=34, RAS-, N=75) were incubated with dose-range Trametinib in liquid culture. Cell viability was determined after 48h using a luminescent reagent and half-maximal effective concentrations (EC_{50}) values of Trametinib were calculated for each sample.*:$p<0.05$. **J.** BEAT AML data on inhibitory concentration 50 (IC_{50}) values of primary AML cells exposed to the MEK inhibitors trametinib or selumetinib dependent on their RAS+ status as indicated.

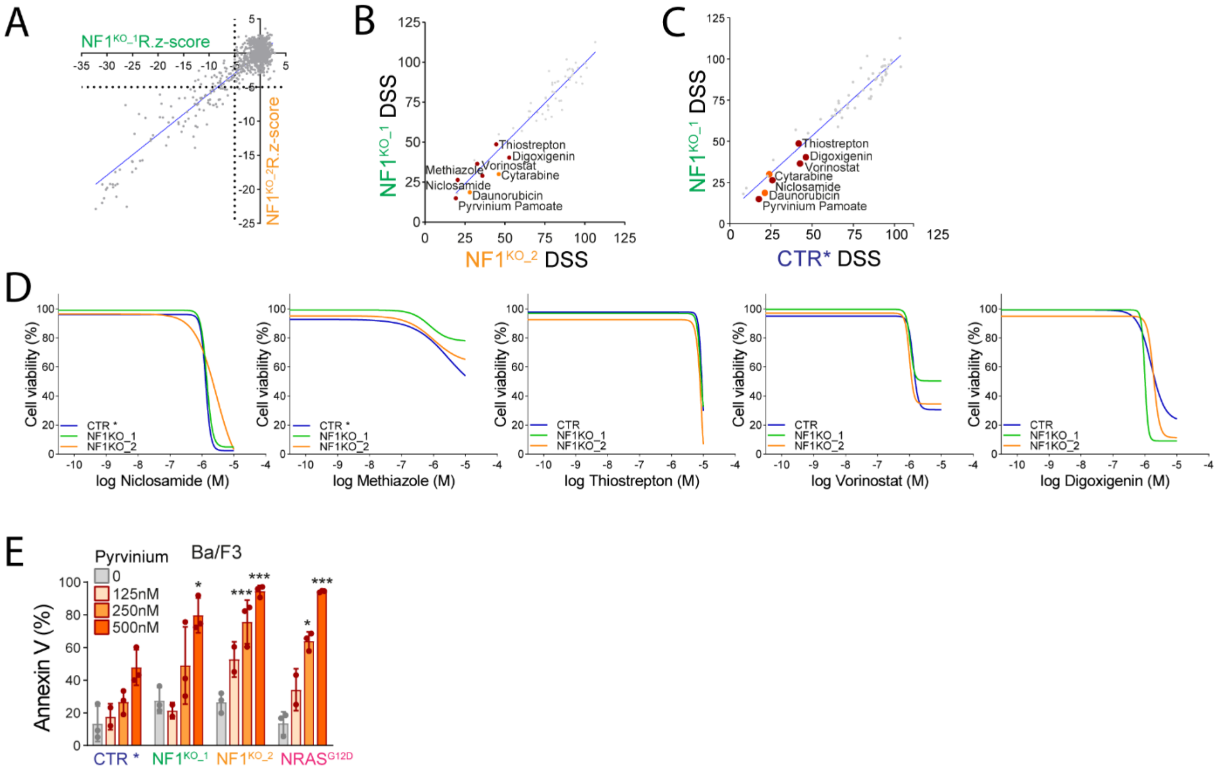

Supplemental Figure 4. Identification of pyrvinium pamoate as anti-leukemic compound active in RAS+ AML. A-B. Ten micromolar screen with 1280 compounds using the CellTiter-Glo® cell viability reagent after 72h of incubation. Robust z-scores (RZ-scores) for each compound identified by a single dot are correlated between $NF1^{KO\_1}$ and $NF1^{KO\_2}$ cells. A dashed line indicate RZ-score ≤-5. **B-C.** Secondary dose-range experiments with the top-60 compounds. Correlation between $NF1^{KO\_1}$ and $NF1^{KO\_2}$ (**B.**), and $NF1^{KO\_1}$ and CTR (in the presence of GM-CSF as indicated by *, **C.**) secondary screens based on drug sensitivity score (DSS) values. Top height compounds are highlighted. **D.** Individual dose-range experiments in CTR*, $NF1^{KO\_1}$ and $NF1^{KO\_2}$ TF-1 cells with five out of the six top-compounds (Niclosamide, Methiazole, Thiostrepton, Vorinostat and Digoxigenin) identified by the secondary screen. **E.** Annexin V flow cytometry binding assays in CTR, $NF1^{KO\_1}$, $NF1^{KO\_2}$ or $NRAS^{G12D}$ Ba/F3 cells incubated with different concentrations of pyrvinium as indicated.

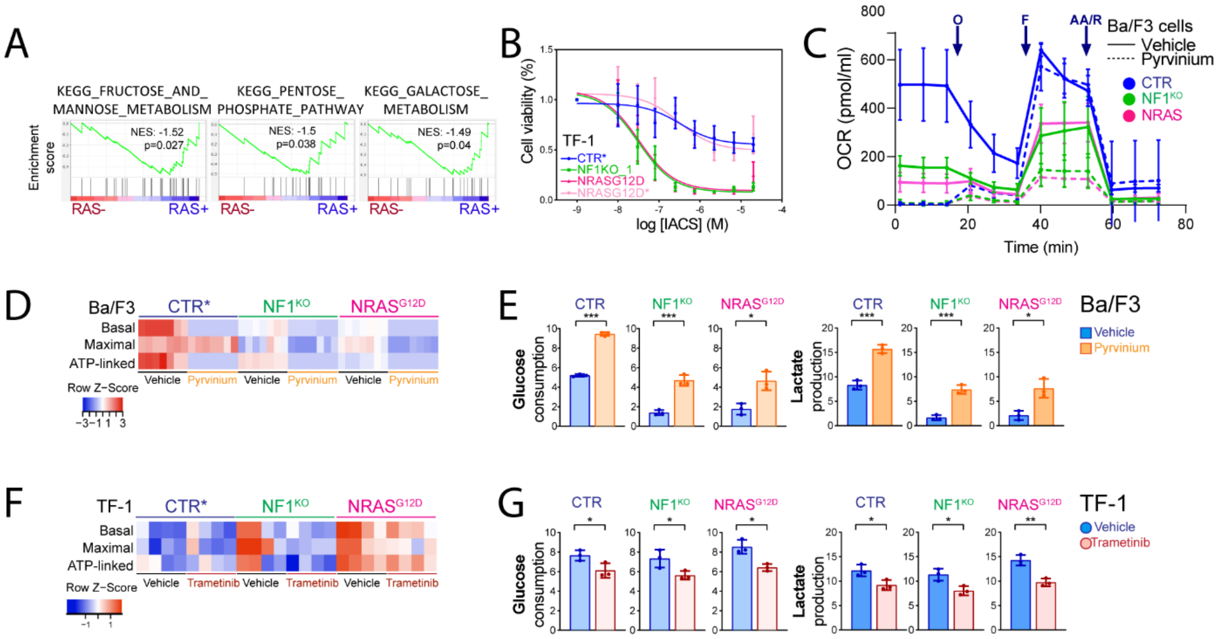

Supplemental Figure 5. Pyrvinium pamoate targets mitochondrial respiration in AML. A. Analysis of gene signature enrichment in the transcriptome of RAS+ and RAS- primary AML samples as described in Figure 1 with a focus on metabolic pathways. **B.** Dose-response assays of the complex I mitochondrial electron transport chain inhibitor IACS-010759 in CTR, NF1^{KO-1} and NRAS^{G12D} TF-1 cells Cultured without or with GM-CSF as indicated by *) using the ATPlite cell viability reagent. **C-D.** CTR, NF1^{KO-1} or NRAS^{G12D} Ba/F3 cells were incubated during 6h with vehicle (CTR in the presence of IL3) or 250nM pyrvinium pamoate in bioenergetic analysis assays measuring the oxygen consumption rate (OCR). **C.** OCR evolution dependent on time. O: olygomycin; F: FCCP; AA/R: antimycin A / rotenone. **D.** Heatmap representation of calculated basal, maximal and ATP-linked OCR. **E.** Glucose consumption and lactate production in CTR*, NF1^{KO} and NRAS^{G12D} cells incubated with vehicle or 250nM pyrvinium for 24h. **F.** CTR*, NF1^{KO-1} or NRAS^{G12D} TF-1 cells were incubated during 6h with vehicle (CTR) or 25nM trametinib in bioenergetic analysis assays measuring OCR. Results are presented as a heatmap of calculated basal, maximal and ATP-linked OCR. **G.** Glucose consumption and lactate production in CTR*, NF1^{KO} and NRAS^{G12D} TF-1 cells incubated with vehicle or 25nM trametinib for 24h. *: indicate that Ba/F3 and TF-1 CTR cells are incubated with IL-3 or GM-CSF, respectively. Vertical bars indicate standard deviations. *:$p<0.05$, **:$p<0.01$, ***:$p<0.001$.

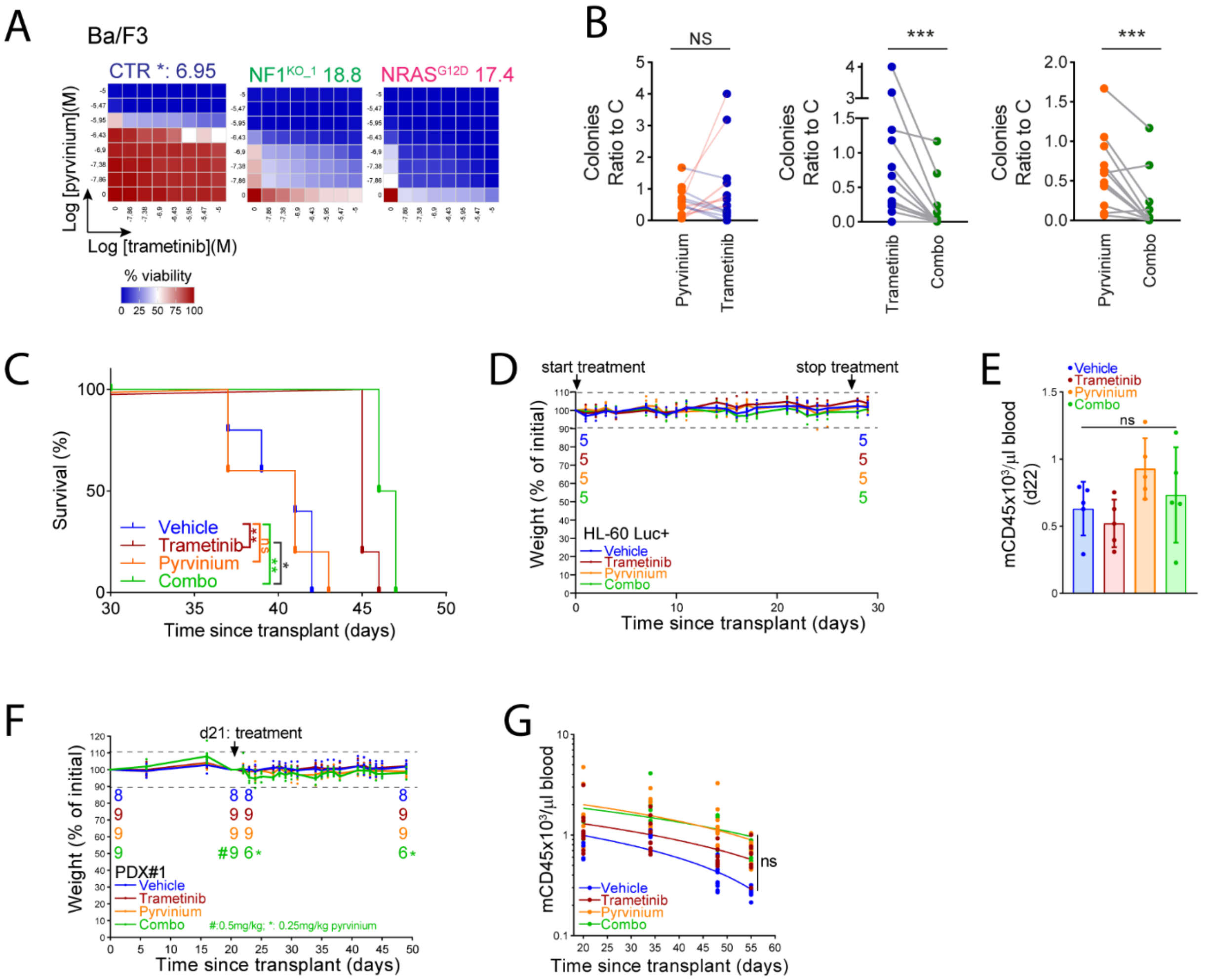

Supplemental Figure 6. Synergy between the MEK inhibitor trametinib and pyrvinium pamoate in RAS activated cells. A. Combination drugs dose-range assays in CTR (with IL3), NF1 KO and NRASG12D Ba/F3 cells incubated with pyrvinium and/or trametinib for 48h. Heat maps provided the combined results of three independent experiments. Synergy scores calculated by the SynergyFinder software (24) from these matrixes are provided for each condition at the top of the heatmap. **B.** L-CFU assays in RAS+ primary AML samples incubated with vehicle, 50nM trametinib, 250nM pyrvinium pamoate or trametinib/pyrvinium combination (combo) during 7 days. Two-by-two comparisons between pyrvinium/trametinib, trametinib/combo and pyrvinium/combo represented with a connecting line between each condition for each sample. Statistical analysis was performed using a Wilcoxon matched-pairs signed rank test. **C.** HL-60 cell line transduced with a vector expressing luciferase (HL-60 Luc+) was injected to immunodeficient NSG recipient mice. Treatment with vehicle, 0.25mg/kg/d trametinib (oral gavage), 0.5mg/kg pyrvinium (intraperitoneal injection) or combination of trametinib and pyrvinium started the day of transplantation (N=5 mice per treatment group). **D.** Evolution of mice weight during the HL60 luc+ CLDX experiment. A dashed line indicates the 10% up and down margins surrounding the initial weight (100%). The number of mice at the beginning and at the end of the experiment are indicated following the color code for each experimental group. **E.** Quantification of murine hematopoietic cells using flow cytometry mCD45 staining at day 22 of the HL60 luc+ CLDX experiment. **F.** Evolution of mice weight during the PDX#1 experiment. A dashed line indicates the 10% up and down margins surrounding the initial weight (100%). The number of mice at the beginning and at the end of the experiment are indicated following the color code for each experimental group. As three mice died at day two of the treatment in the combo group, pyrvinium dose was reduced from

0.5 to 0.25mg/kg/d, as indicated, without further toxicity problem during the further three weeks of treatment. **G.** Quantification of murine hematopoietic cells using flow cytometry mCD45 staining at different time points of the PDX#1 experiment. *: indicate that Ba/F3 CTR cells are incubated with IL-3. Vertical bars indicate standard deviations. ns: not significant. ***:p<0.001.

Supplemental Table 1. RAS mutations found in AML cell lines. Targeted DNA sequencing was performed on a panel of human AML cell lines. NRAS or KRAS mutations may be absent or present, with details of nucleotide substitution and amino acid modification.

**Cell line**	***NRAS* or *KRAS* mutation**
TF-1	*NRAS* Q61P 39% c.182A>C
MO7-e	absent
UT-7	*NRAS* G12C 26% c.34G>T
KASUMI	
THP-1	*NRAS* G12D 65% c.35G>A
HL-60	*NRAS* Q61L 53% c.182A>T
MOLM-14	absent
OCI-AML2	absent
OCI-AML3	*NRAS* Q61L 100% c.182A>T
SKM-1	*KRAS* K117N 100% c.351A>C
K562	absent

Supplemental Table 2. Repartition of myeloid neoplasm related gene mutations dependent on the RAS mutational status in AML samples analyzed by RNAseq.

	RAS+	RAS-	p-val
All cases	24	30	
ASXL1	8	5	0.2
BCOR	2	3	0.99
DNMT3A	4	8	0.52
EZH2	4	3	0.68
IDH2	3	9	0.19
RUNX1	6	5	0.5
SRSF2	5	4	0.49
STAG2	5	2	0.22
TET2	5	5	0.73
TP53	2	4	0.68

Supplemental Table 3. Repartition of myeloid neoplasm related gene mutations dependent on the RAS mutational status. TKD: tyrosine kinase domain mutation. P-values are considered significant if <0.05 and only significant values are provided.

**Gene**	**RAS+ (N, %)**	**RAS- (N, %)**	**P-value**
ASXL1	16 (31%)	13(17%)	
BCOR	6 (11.7%)	10 (13.1%)	
CEBPA	3 (5.8%)	4 (5.3%)	
DNMT3A	10 (19.6%)	16 (21%)	
EZH2	8 (15.7%)	4 (5.3%)	
*FLT3*_TKD	3 (5.8%)	4 (5.3%)	
IDH1	4 (7.8%)	2 (2.6%)	
IDH2	4 (7.8%)	20 (26.3%)	0.011
PHF6	1 (2%)	7 (9.2%)	
RUNX1	12 (23.5%)	11 (14.5%)	
SF3B1	2 (4%)	4 (5.3%)	
SRSF2	9 (17.6%)	14 (18.4%)	
STAG2	8 (15.7%)	7 (9.2%)	
TET2	10 (19.6%)	11 (14.5%)	
TP53	12 (23.5%)	15 (19.7%)	
Complex Karyotype	16 (31%)	16 (21%)	

Supplemental Methods

Bio-informatics analysis of DNA sequencing data

Base calls were generated by the Torrent Suite™ Software (v. 5.6) using the included variant caller with an additional plug-in (Life Technologies). The .bam and .vcf files were used for analysis. Detection of single nucleotide variations (SNVs) and short insertions/deletions from the BAM files was performed using the Torrent Suite Variant Caller (TSVC) plugin from the Torrent Suite Software v5.0.4 (Thermo Fisher Scientific). The .vcf files were annotated with the Ion reporter software (Life Technologies) and processed for a second analysis of the indexed files using the NextGENe software (Softgenetics, State College, USA). Results were compared to select abnormalities that will be further considered. Filtered candidate variants listed in TSVC files were then annotated, ranked, and interpreted using the Polydiag suite (Bioinformatics Department, Paris-Descartes University, Paris, France). Moreover, aligned reads from .bam files were visualized using the Integrative Genomics Viewer v2.3 from the Broad Institute (Cambridge, MA, USA). Assessment of variants implication was performed based on population databases (dbSNP and GnomAD), mutation databases (COSMIC), and predictions software (Alamut, mutation taster, OncoKB, and Cancer Genome Interpreter). For hierarchical clustering, we used the top 1000 most variable genes using nclust tool (https://gitlab.com/pwirapati/nclust) The heatmap of the clustering was visualized using the Heatmap () function from the ComplexHeatmap R package [3].

Microarrays

Double strand cDNA was used for in vitro transcription with T7 RNA polymerase and 5.5µg of Sens Target DNA was fragmented and labelled with biotin. The cDNA were then hybridized to GeneChip® Clariom S Human (Affymetrix) at 45°C for 17 hours, then washed on the fluidic station FS450 (Affymetrix), and scanned using the GCS3000 7G (Thermo Fischer Scientific). Scanned images were then analyzed with Expression Console software (Affymetrix) to obtain raw data (.cel files) and metrics for quality controls. Raw fluorescence intensity values were normalized using Robust Multi-array Average (RMA) algorithm in R to generate the normalized data matrix by performing background correction, quantile normalization and log2 transformation of raw fluorescence intensity values of each gene. All quality controls and statistics were performed using Partek® Genomics Suite software (Partek, St. Louis, MO, USA). Data were normalized using custom brainarray CDF files (v20 ENTREZG). To identify differentially expressed genes, we applied a classical analysis of variance (ANOVA) with an FDR permutation-base for each gene. We created a new matrix with only the significant ANOVA site and performed Z-scoring of rows. Hierarchical clustering by Pearson's dissimilarity and average linkage and principal components analysis (PCA) were conducted in an unsupervised fashion to control for experimental bias or outlier samples. We set a filter for those genes that displayed at least a ≥1,5 or ≤-1,5-fold difference in expression between groups and achieved an FDR of <0.05. Data were then

interrogated for evidence of biologic pathway dysregulation using Gene set enrichment analysis (GSEA, Broad Institute). Enrichment rates were considered significant when the P-value <0.05 and the FDR ≤0.1.

RNA-seq quantification

Paired-end reads were aligned to the human reference transcriptome "Homo_sapiens.GRCh38.cdna.all.fa.gz" using Salmon 1.3.0. For mapping, we used salmon quant command with parameters like –gcBias, --useVBOpt, --seqBias, and –validateMappings, correcting specific biases in the sequenced reads and increasing the sensitivity and specificity of the mapping. After mapping, R (R version R version 4.0.2) packages tximport (version 1.14.0) and DESeq2 (version 1.26.0) were used to summarize transcript-level estimate to the gene-level estimates. Further, we used the raw gene-level abundance estimates from salmon to perform to identify differentially expressed genes.

Differential gene expression analysis

Differential gene expression analysis was performed using an R Bioconductor software package edgeR [4]. First, we filtered the genes with a meager count using an in-built function in edgeR filterByExpr(), and the library size was recomputed. Following this, the normalization factors were estimated using the calcNormFactors() function, and the per gene dispersion was assessed using the estimateDisp() function in edgeR. Here, a design formula (linear model, ~Rasopathy) was provided for dispersion calculation. Finally, the negative binomial generalized linear model was fit using glmFit() function, and likelihood ratio test glmLRT() was used to test for differential expression. We assessed the differentially expressed genes between the Rasopathy-Yes vs. Rasopathy-No conditions. The topTags() function was used to extract the top-ranked genes, and the results were presented as a volcano plot using an R Bioconductor package EnhancedVolcano. We also performed gene set enrichment analysis [5] using the oncogenic signature gene set in which we manually added several gene sets from recent literature [6–9].

Lentivirus production and cell line infections

Lentivirus production and cell line infections were done as previously described [10]. Briefly, we used 293-T packaging cells to produce all of the constructed recombinant lentivirus through co-transfection of these cells with the packaging plasmids pMD2.G and psPAX2 encoding lentiviral proteins (Gag, Pol, and Env) using Lipofectamine 2000 Transfection Reagen (Thermo Fischer Scientific, Waltham, Massachusetts, US). Supernatants were collected and ultracentrifuged for 48 h after transfection over two consecutive days, and then stored at -80°C. AML cell lines were seeded at $2x10^6$/ml and 10µl of lentiviral supernatants were added for 24h. Cells were further selected with puromycin, or cell sorted with an ARIA 3 cytometer in case of GFP or mCherry expression as selection marker.

TF-1 differentiation

Cells were washed 3 times in PBS to remove GM-CSF, and then cultured 7 days with 2 IU/mL EPO. Cells were spin down to collect pellets in which color change from white to purple reflected hemoglobinization.

Trypan Blue dye exclusion assay

The Trypan Blue dye (Sigma Aldrich) exclusion assay was used to determine the number of viable cells present in the cell suspension. A Malassez counting chamber was filled with the cell suspension mixed with the dye. Cells were then visually examined and counted under a microscope: cells taking up the dye were considered dead and cells excluding the dye were considered alive.

Immunohistochemistry

Femurs, tibias and spleens of mice were fixed for 24h in 4% paraformaldehyde. Decalcification was carried out using 15% formic acid at 4°C for 4h, followed by a second fixation in 4% paraformaldehyde during 24h. Samples were paraffin embedded and then sliced using a microtome. Four µm thick serial sections were analyzed by immunohistochemistry using anti-phospho-ERK antibody (#4370, Cell Signaling Technology, Danvers, Massachusetts, US) with Immunohistochemistry Application Solutions Kit (Rabbit) (#13079, Cell Signaling Technology) according to the manufacturer's instructions. For antigen retrieval, slides were heated in citrate buffer (10mM sodium citrate buffer pH 6,0) for 10 min. Anti-phospho-ERK antibody was solubilized at 1/200 in an antibody diluent solution (#8112, Cell Signaling Technology) and incubated over night at 4°C. Detection of primary antibodies was carried out using the Signal Stain Boost IHC Detection Reagent (#8114, Cell Signaling Technology) and Signal Stain DAB Substrate (#8059, Cell Signaling Technology) based on conversion of diaminobenzidine to a dye with multimeric horseradish peroxidase (HRP). Sections were counterstain with Hematoxylin. Images were acquired and processed using the slide scanner and software Zeiss Axioscan.Z1 (Carl Zeiss AG, Oberkochen, Germany).

Prestwick chemical library® (PCL) screen

<u>Primary screening</u>

PCL compounds were added 24h after cell seeding: 2µL/well of each compound (from 10 mM stock plates) were diluted in 8µL of DMSO in order to obtain 2mM/well stock plates. Next, 2µl/well at 2mM were mixed in a predilution plate containing 78µL/well of cell media, and 10µL of this solution were dispensed into each 384-cell plate well using the MultiChannel Arm™ 384 (TECAN, Männedorf, Switzerland), to a final concentration of 10µM and 0.5% of DMSO. The plates were incubated for 72h at 37°C in 5% CO2 before submitted to CellTiter-Glo® 2.0 cell viability assay. Biological duplicates were performed.

Secondary screening

We selected the top 60 hits from our primary screen to perform a set of dose-response experiments using the same workflow. We performed three independent experiments using 8 consecutive three-fold dilutions from 10^{-6}M to 4.57 x 10^{-9}M. Both primary and secondary screens were performed on the same batches of viably frozen cells and at the same passage stage (four passages from thawing).

Data processing

Values of all plates were visually inspected for systematic bias (i.e., edge effects). Raw luminescence intensity values were log2 transformed, then median polishing was applied to progressively corrects columns, rows, and entire plates by subtracting their median, repeating until convergence of values. Column and row corrections were computed separately for each experimental replicates, but across all plates within the replicate in combination. Hits were identified by the robust Z-score method under the assumption that most compounds are inactive and can serve as controls as follows: sample median and median absolute deviation (MAD) were calculated from the population of screening data points (named as sample) and used to compute robust Z-scores according to the formula:

"$RZ{-}score$"=($x{-}sample\ median$) / (1.4826× MAD), where x corresponds to the compound-treated data point and MAD is the median of the absolute deviation from the median of the tested wells. A compound was identified as a hit if the RZ-score was < -2 or > 2 pointing in the same direction in both replicates. Compounds having a RZ-score < -2 corresponds to those considered reducing cell viability. The same analysis pipeline was applied to each cell lines tested. Final values correspond to the mean RZ-score for each compound.

In dose-response experiments, compound activity was normalized on a per-plate basis by dividing the value in each well by the median value of the control wells (100% cell viability). For each compound, a four parameters log-logistic model was then fitted on the pooled replicate data with the R package drc. Compound activity was then summarized by computing a Drug Sensitivity Score (DSS, modified from [11]), the area under the curve normalized by the area of an inactive compound (100% viability at all doses). Finally, compounds were scored by calculating their median dose-effect (effective dose 50, ED50) x DSS value and the top-10 compounds among which were the classical AML chemotherapies cytarabine and daunorubicin were selected for further assays.

Synergistic cell viability assays

We performed dose-range experiments of trametinib and pyrvinium single-agents or combination and assessed cell viability after 48h using the Uptiblue® reagent. We used the SynergyFinder online software to calculate synergy scores computed using the zero interaction potency (ZIP) model. All experiments were done three times separately and pooled data were analyzed.

References

1 Papaemmanuil E, Gerstung M, Bullinger L, Gaidzik VI, Paschka P, Roberts ND *et al.* Genomic Classification and Prognosis in Acute Myeloid Leukemia. *N Engl J Med* 2016; **374**: 2209–2221.

2 Wang T, Yu H, Hughes NW, Liu B, Kendirli A, Klein K *et al.* Gene Essentiality Profiling Reveals Gene Networks and Synthetic Lethal Interactions with Oncogenic Ras. *Cell* 2017; **168**: 890-903.e15.

3 Gu Z, Eils R, Schlesner M. Complex heatmaps reveal patterns and correlations in multidimensional genomic data. *Bioinformatics* 2016; **32**: 2847–2849.

4 Robinson MD, McCarthy DJ, Smyth GK. edgeR: a Bioconductor package for differential expression analysis of digital gene expression data. *Bioinformatics* 2010; **26**: 139–140.

5 Subramanian A, Tamayo P, Mootha VK, Mukherjee S, Ebert BL, Gillette MA *et al.* Gene set enrichment analysis: a knowledge-based approach for interpreting genome-wide expression profiles. *Proc Natl Acad Sci U S A* 2005; **102**: 15545–15550.

6 Farge T, Saland E, de Toni F, Aroua N, Hosseini M, Perry R *et al.* Chemotherapy-Resistant Human Acute Myeloid Leukemia Cells Are Not Enriched for Leukemic Stem Cells but Require Oxidative Metabolism. *Cancer Discov* 2017; **7**: 716–735.

7 Wagle M-C, Kirouac D, Klijn C, Liu B, Mahajan S, Junttila M *et al.* A transcriptional MAPK Pathway Activity Score (MPAS) is a clinically relevant biomarker in multiple cancer types. *NPJ Precis Oncol* 2018; **2**: 7.

8 Way GP, Sanchez-Vega F, La K, Armenia J, Chatila WK, Luna A *et al.* Machine Learning Detects Pan-cancer Ras Pathway Activation in The Cancer Genome Atlas. *Cell Rep* 2018; **23**: 172-180.e3.

9 Uhlitz F, Sieber A, Wyler E, Fritsche-Guenther R, Meisig J, Landthaler M *et al.* An immediate-late gene expression module decodes ERK signal duration. *Mol Syst Biol* 2017; **13**: 928.

10 Grenier A, Sujobert P, Olivier S, Guermouche H, Mondésir J, Kosmider O *et al.* Knockdown of Human AMPK Using the CRISPR/Cas9 Genome-Editing System. *Methods Mol Biol* 2018; **1732**: 171–194.

11 Yadav B, Pemovska T, Szwajda A, Kulesskiy E, Kontro M, Karjalainen R *et al.* Quantitative scoring of differential drug sensitivity for individually optimized anticancer therapies. *Sci Rep* 2014; **4**: 5193.
